# Synthesis and Study of Multifunctional Cyclodextrin–Deferasirox Hybrids

**DOI:** 10.1002/cmdc.201900334

**Published:** 2019-06-24

**Authors:** Jose Miguel Gascon, Valentina Oliveri, Andrew McGown, Ecem Kaya, Yu‐Lin Chen, Carol Austin, Martin Walker, Frances M. Platt, Graziella Vecchio, John Spencer

**Affiliations:** ^1^ Department of Chemistry School of Life Sciences University of Sussex Falmer Brighton East Sussex BN1 9QJ UK; ^2^ Dipartimento di Scienze Chimiche Università degli Studi di Catania Viale A. Doria 6 95125 Catania Italy; ^3^ Department of Pharmacology University of Oxford Mansfield Road Oxford OX1 3QT UK; ^4^ Pharmaceutical Science King's College London Franklin Wilkins Building London SE1 9NH UK; ^5^ Eurofins Selcia Drug Discovery Fyfield Business & Research Park Fyfield Road, Ongar Essex CM5 0GS UK

**Keywords:** antioxidant, deferasirox, iron complexes, Niemann–Pick, protein aggregation

## Abstract

Metal dyshomeostasis is central to a number of disorders that result from, inter alia, oxidative stress, protein misfolding, and cholesterol dyshomeostasis. In this respect, metal deficiencies are usually readily corrected by treatment with supplements, whereas metal overload can be overcome by the use of metal‐selective chelation therapy. Deferasirox, 4‐[(3*Z*,5*E*)‐3,5‐bis(6‐oxo‐1‐cyclohexa‐2,4‐dienylidene)‐1,2,4‐triazolidin‐1‐yl]benzoic acid, Exjade, or ICL670, is used clinically to treat hemosiderosis (iron overload), which often results from multiple blood transfusions. Cyclodextrins are cyclic glucose units that are extensively used in the pharmaceutical industry as formulating agents as well as for encapsulating hydrophobic molecules such as in the treatment of Niemann–Pick type C or for hypervitaminosis. We conjugated deferasirox, via an amide coupling reaction, to both 6^A^‐amino‐6^A^‐deoxy‐β‐cyclodextrin and 3^A^‐amino‐3^A^‐deoxy‐2^A^(*S*),3^A^(*S*)‐β‐cyclodextrin, at the upper and lower rim, respectively, creating hybrid molecules with dual properties, capable of both metal chelation and cholesterol encapsulation. Our findings emphasize the importance of the conjugation of β‐cyclodextrin with deferasirox to significantly improve the biological properties and to decrease the cytotoxicity of this drug.

## Introduction

Chelation therapy is considered as one treatment option to decrease the toxic effects of metal ions in humans. Besides the removal of toxic foreign metals, chelation therapy is also used to lower the levels of essential metals in cases of copper or iron overload disorders as in Wilson's disease and primary and secondary hemochromatosis.^1^, ^2^ Moreover, the use of chelating agents has also been proposed for neurodegenerative disorders related to oxidative stress, and the disruption of metal and cholesterol homeostasis.^3^, ^4^ Deferasirox (**3**, Exjade, ICL670) is an orally available, once‐daily, clinical iron chelator commonly used to treat hemosiderosis resulting from multiple blood transfusions, non‐transfusion‐dependent thalassemia (NTDT), sickle‐cell anemia, or myelodysplastic syndromes (MDS).^5^ The development of chelating agents for iron, which currently includes desferal, deferiprone, and deferasirox, is challenging given the various side‐effect drawbacks associated with these agents.^6^, ^7^, ^8^ In particular, nephrotoxicity is one of the most frequent adverse effects of iron chelation treatment.^9^ In an attempt to overcome these limitations, different strategies have been proposed, viz. covalent modification of the deferasirox scaffold^10^ and noncovalent inclusion in a cyclodextrin cavity.^11^

We previously conjugated metal chelators such as deferiprone and clioquinol with β‐cyclodextrin (CyD).^12^, ^13^ Studies on these conjugates indicated that CyD conjugation improves some features of the chelators and makes them multifunctional.^14^, ^15^ CyDs have been studied as artificial chaperones and anti‐aggregant agents.^16^, ^17^ Methyl‐β‐CyD has been reported to reduce α‐synuclein (αSyn) accumulation, a protein implicated in Parkinson's disease (PD).^18^ Also, some authors have reported that dietary CyDs can influence metallothionein mRNA levels in rats.^19^ This may cooperate with the chelating ability of the CyD moiety, as metallothioneins play a prominent role in metal homeostasis. Finally, the CyD cavity can be available to include exogenous drugs as well as endogenous cholesterol and lipids that are also involved in neurodegenerative diseases.

**Figure 1:**
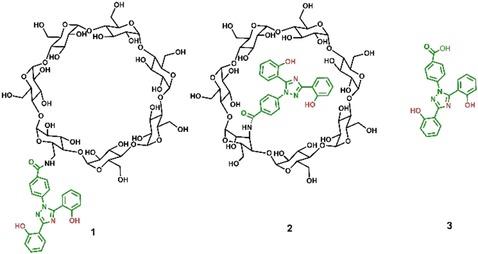
Structures of the derivatives in this study.

Several studies have investigated the effect of cholesterol‐reducing agents such as CyDs on neurodegenerative pathologies.^20^, ^21^ Trappsol® [hydroxypropyl‐β‐cyclodextrin (HPBCD)] is currently under clinical trials for the treatment of Niemann–Pick type C (NPC), a lysosomal lipid storage disorder characterized by an accumulation of lipids such as cholesterol. This inspired us to synthesize and investigate new conjugates of CyD with deferasirox. Herein we report the synthesis and characterization of 4‐[3,5‐bis(2‐hydroxyphenyl)‐1*H*‐1,2,4‐triazol‐1‐yl]‐*N*‐[6^A^‐amino‐6^A^‐deoxy‐β‐cyclodextrin]benzamide (**1**, Figure 1) and 4‐[3,5‐bis(2‐hydroxyphenyl)‐1*H*‐1,2,4‐triazol‐1‐yl]‐*N*‐[3^A^‐deoxy‐3^A^‐amino‐β‐cyclodextrin]benzamide (**2**, Figure 1), as 3‐ and 6‐monofunctionalized β‐cyclodextrins may have significantly different chemical and biological properties owing to their different structures. Given the multifactorial nature of neurodegenerative disorders, we evaluated the ability of the new CyD conjugates to act on multiple targets. Hence, we studied the antioxidant activity of the deferasirox conjugates, the stability of their iron(III) complexes, and the ability to inhibit metal‐induced αSyn aggregation. We also evaluated the cytotoxicity of **1** and **2** in vitro and their effects in NPC cell models. Deferasirox (**3**) was also investigated for comparative purposes. The chemical and biological properties of the conjugates confirm that conjugation with the CyD cavity is a promising strategy to design new nontoxic multifunctional molecules.

## Results and Discussion

### Synthesis and characterization

Considering the pharmacological properties of **3** and the ability of CyDs to act as carriers, anti‐aggregants, and neuroprotective agents, we envisaged a simple reaction via the carboxylic group of **3** to obtain novel CyD conjugates. Amide condensation was thus performed between **3** and 6^A^‐amino‐6^A^‐deoxy‐β‐cyclodextrin or 3^A^‐amino‐3^A^‐deoxy‐2^A^(*S*),3^A^(*S*)‐β‐cyclodextrin in the presence of suitable activating agents to obtain the 6‐monofunctionalized **1** and the 3‐monofunctionalized **2** isomers, respectively.

Such conjugation was confirmed by mass spectrometry (MS). ESI‐MS data of conjugates **2** and **1** show two peaks resulting from singly charged (*m*/*z* 1511.4 [*P*+Na]^+^) and doubly charged ions (*m*/*z* 764.2 [*P*+H+K]^2+^). 6‐Functionalized and 3‐functionalized derivatives have significant differences due to the different synthetic routes followed to obtain the amino‐CyD used in the amide condensation reaction. Because of a chair flip of the modified altrose unit in the 3‐functionalized CyD derivatives, the cavity is elliptically distorted, whereas functionalization at the 6‐position does not dramatically influence the CyD cavity, according to the literature.^22^, ^23^ Thus, the kind of functionalization strategy used to obtain the CyD conjugates may confer different properties to the resulting conjugates.^24^

The new compounds were further characterized by ^1^H and ^13^C NMR spectroscopy. The 1D spectra of the products were assigned using 2D experiments (COSY, TOCSY, HSQC, HMBC, and ROESY; Figures S1–S6 in the Supporting Information). The NMR spectra of **1** and **2** further confirm ligand conjugation, as they display signals due to the **3** and CyD moieties, the former resonating in the aromatic region. Other diagnostic signals of these CyD conjugates include those of the carbonyl groups at *δ*=166.0 and 165.4 ppm in the ^13^C NMR spectrum of **1** and **2**, respectively.

**Figure 2:**
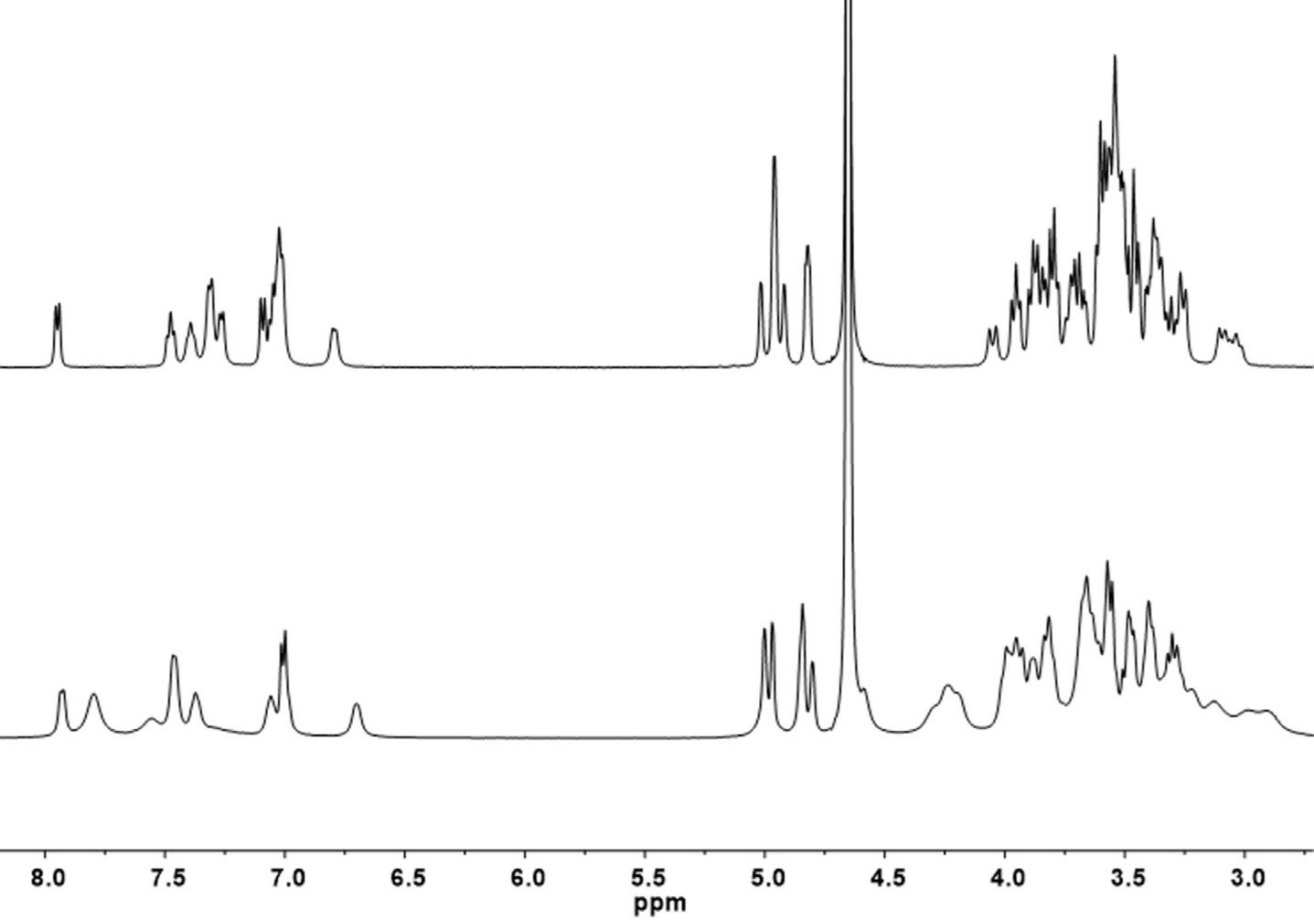
^1^H NMR spectra of **1** (top) and **2** (bottom) in D_2_O at 500 MHz.

As for **1**, chemical shifts corresponding to H‐1 protons (Hs‐1) of the CyD moiety are divided into four groups (Figure 2) upon the functionalization that made them inequivalent. Moreover, H‐6 protons (Hs‐6) of the CyDs are spread as a consequence of the presence of the aromatic moiety linked to the CyD rim. In particular, the H‐6A protons appear at *δ*=4.05 and 3.04 ppm because of the aromatic ring current effect as reported for other aromatic CyD derivatives.^25^

**Figure 3:**
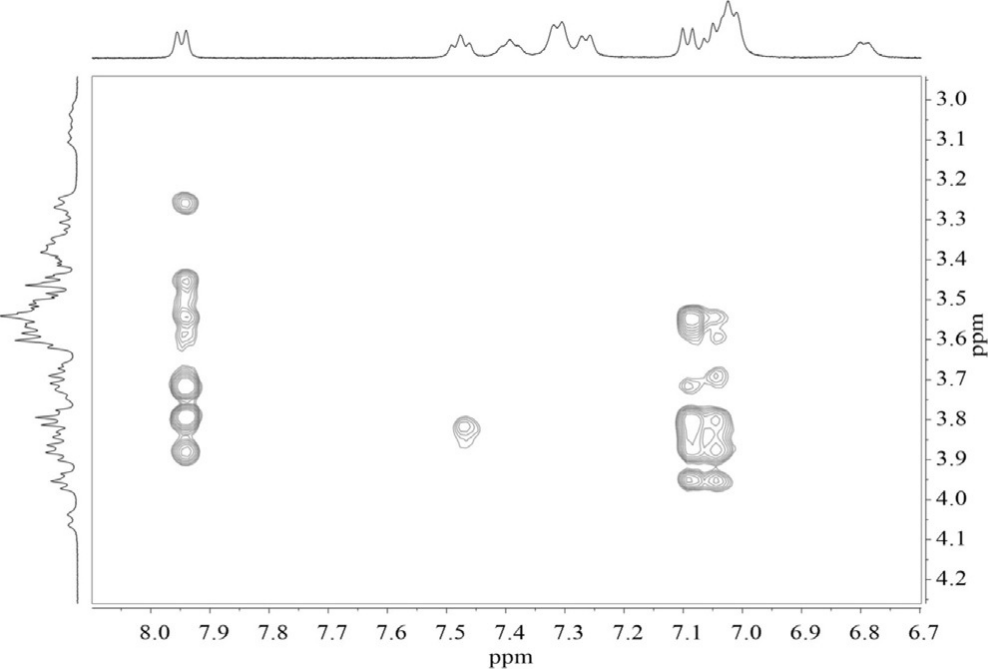
ROESY spectrum of **1** in D_2_O at 500 MHz.

Analysis of the ROESY spectrum (Figure 3) reveals the presence of through‐space proximities between an aromatic ring of the **3** moiety and Hs‐3 and Hs‐5 of the CyD. Because these protons of the CyD are pointed toward the interior of the cavity, the formation of an inclusion complex of the pendant with the CyD cavity can be hypothesized. In particular, intense cross‐peaks were detected between H‐13, H‐14, H‐15, and H‐16 of the aromatic ring **a** and Hs‐3 and Hs‐5 of the CyD. ROESY data suggest that the phenolic ring **a** is inside the CyD cavity.

**Figure 4:**
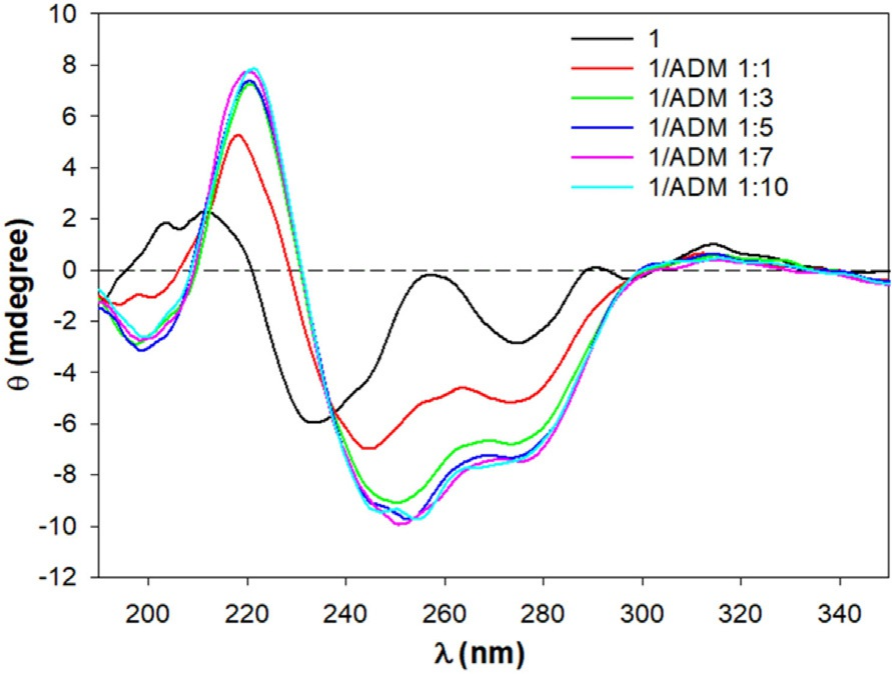
CD spectra of **1** (50 μm) in the presence of increasing concentrations of ADM (50–500 μm).

Moreover, the NMR spectrum did not change with an increase in the concentration of **1**, suggesting the intramolecular interaction and thus self‐inclusion of the pendant. This compound was also characterized by CD spectroscopy to further investigate the interaction of the **3** moiety with the β‐CyD cavity. The CD spectrum of **1** at pH 6.8 shows two positive bands at 211 and 315 nm and two more intense negative bands at 233 and 275 nm. These bands are in the absorption region of the aromatic rings and are due to the dipole–dipole coupling between the **3** moiety and the β‐CyD cavity. This behavior has been generally reported for functionalized CyDs, and it is due to the interaction of the functionalizing moiety^26^ with the CyD. Furthermore, the CD spectrum is strongly influenced by the presence of 1‐adamantanol (ADM), a well‐known high‐affinity guest of the CyD cavity, in keeping with the self‐inclusion (Figure 4 and Figure S7). The intensity and absorption maximum wavelength of the band's changes with increasing concentrations of ADM suggesting a modification of the orientation of the aromatic pendant concerning the CyD cavity.

Analogously to **1**, the self‐inclusion of the aromatic ring **a** can be proposed for the 3‐functionalized conjugate by NMR and CD data. The ^1^H NMR spectrum of **2** shows that the functionalization splits out the H‐1 protons of the CyD into five groups (Figure 2). In particular, H‐1A of the functionalized sugar of the CyD resonates at 4.58 ppm. The synthetic strategy followed to functionalize CyDs at the 3‐hydroxy group leads to configuration inversions of C2 and C3 and an altrose unit (ring A) replaces a glucose unit in the CyD molecule (Figure S8). As a consequence, H‐2A and H‐3A are strongly shifted downfield at 4.30 ppm as typically observed for other 3‐functionalized derivatives.^19^ ROESY spectra display intense NOE‐correlation peaks between aromatic protons and inner CyD protons suggesting the inclusion of ring **a** (Figures S9 and S10).

**Figure 5:**
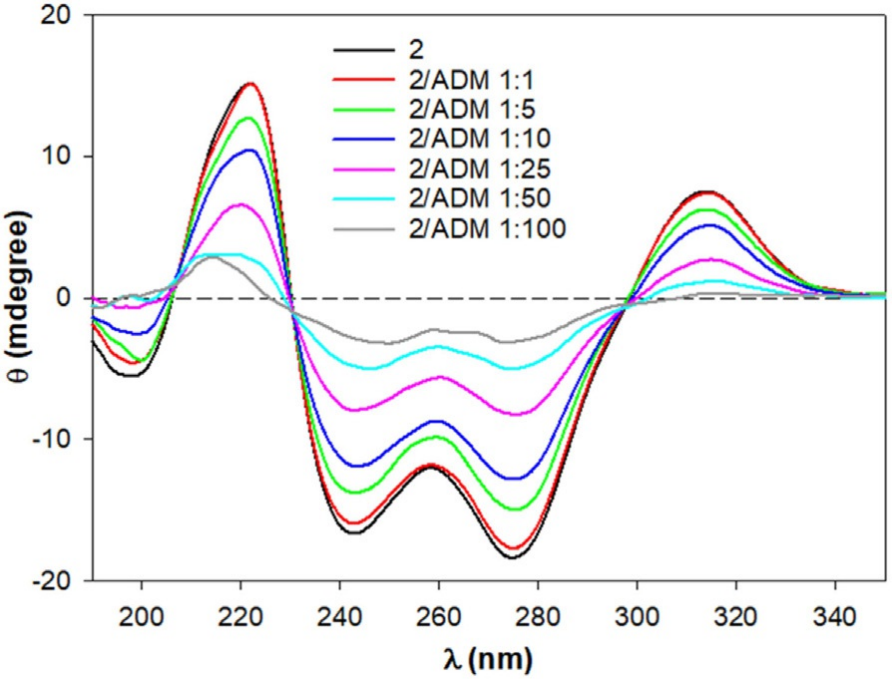
CD spectra of **2** (50 μm) in the presence of increasing concentrations of ADM (50 μm–5 mm).

Finally, further evidence of self‐inclusion of the pendant is provided by CD experiments (Figure 5) in the presence of ADM. CD spectra of **2** show intense dichroic bands at 222, 233, 275, and 315 nm. These bands are associated with the π–π* absorption bands of the aromatic moiety. The differences in CD spectra of **1** and **2** can be anticipated according to the empirical rules that interpret the induced circular dichroism observed for a chromophore inside or outside the CyD cavity.^26^ Compounds **1** and **2** are structural isomers, and the rim of **2** is quite distorted owing to the presence of the altrose ring. For this reason, a different orientation of the **3** moiety in **1** and **2** can explain the different CD spectra. A decrease in the intensity of the dichroic bands of **2** with increasing concentrations of ADM reveals the displacement of the included aromatic ring from the CyD cavity (Figure 5).

### Proton and iron(III) complex stability constants

Ligand **3** possesses a 1,2,4‐triazole framework substituted by two phenolic substituents at positions 3 and 5 and a benzoic acid moiety at position 1. The values of proton/iron(III) stability constants were determined by spectrophotometric titrations (Figures S11–S19) and are listed in Tables 1 and 2.

For protonation stability constants (Table 1), p*K*_a‐carboxy_ corresponds to the deprotonation of the carboxylic group, whereas the values of p*K*_a‐phenol1_ and p*K*_a‐phenol2_ are characteristic of the deprotonation of the phenolic groups. The values determined in this study are consistent with the values reported elsewhere.^27^

**Table 1:** p*K*_a_ values for **3** and the two conjugates (25 °C, 0.1 m KCl) in H_2_O/DMSO solution (molar ratio 1:0.2); repeated measurements can result in about 3 % error.

p*K*_a_	**3**	**1**	**2**
p*K*_a‐carboxy_	4.8	–	–
p*K*_a‐phenol1_	10.3	8.8	9.5
p*K*_a‐phenol2_	12.3	12.1	12.0

In comparison with **3**, both CyD compounds **1** and **2** possess lower p*K*_a_ values associated with the deprotonation of phenolic groups. NMR and CD data suggest the self‐inclusion of the **a** ring of the CyD derivatives, and this behavior could decrease the protonation constant of this phenol group relative to that of **3**. A decrease in the values of the protonation stability constants has been reported for other CyD derivatives and has been attributed to the hydrophobic effects of the CyD cavity.^12^

For iron(III) stability constants, **3** is a tridentate chelating agent, mainly forming a 2:1 chelator/iron(III) complex at physiological pH.^28^ Our data (Table 2) confirm these results and demonstrate that the CyD conjugates form the FeL and FeL_2_ species similarly to **3**. In particular, FeL_2_ is the sole complex species present at physiological pH for both CyD conjugates (Figures S14 and S19; pH 8.4 in H_2_O/DMSO solution at a molar ratio of 1:0.2 is closely equivalent to pH 7.4 in aqueous solution). However, the iron(III) complex stability constants values of **1** and **2** are lower than those of **3**. This trend has been observed for other CyD derivatives and is associated with the steric bulk of the CyD scaffold.

**Table 2:** Overall formation constants (log β_mnq_) of Fe^III^ complexes with **3** and the two conjugates (25 °C, 0.1 m KCl) in H_2_O/DMSO solution (molar ratio 1:0.2); repeated measurements can result in about 3 % error.

log β_mnq_	**3**	**1**	**2**
log β_111_	27.0	–	–
log β_110_	24.5	21.4	21.8
log β_121_	45.3	–	–
log β_120_	39.3	35.3	36.0
pFe^III^_8.4_^[a]^	23.6	22.5	22.7

[a] pFe^III^_8.4_ values were calculated under conditions of [Fe^III^]_total_=1 μm, [ligand]_total_=10 μm, pH 8.4. In H_2_O/DMSO solution (molar ratio 1:0.2), pH 8.4 is closely equivalent to pH 7.4 in aqueous solution.

### Antioxidant activity

Mounting evidence suggests a pivotal role of oxidative stress in metal‐overload diseases^29^ and neurodegenerative diseases,^30^ and hence much effort has been undertaken to target it using new and powerful antioxidants.^31^ Antioxidants such as polyphenols, vitamins A and E have been proposed as therapeutic agents for preventing and decreasing the rate of progression of neurodegenerative diseases.^32^

**Figure 6:**
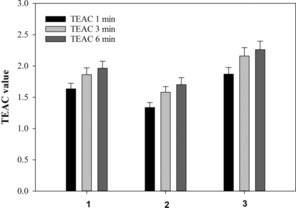
TEAC values at 1, 3, and 6 min for CyD derivatives **1**, **2**, and **3**. The values are expressed as the average of three independent assays, and error bars show standard deviations.

The Trolox equivalent antioxidant capacity (TEAC) assay was used to quantify the antioxidant activity of the CyD derivatives. The capacity of the systems to scavenge ABTS^•+^ at 1, 3, and 6 minutes was compared with Trolox, an analogue of vitamin E, and was thus expressed as TEAC values (Figure 6). The antioxidant activity of **1** is two times higher than that of Trolox and similar to that of **3**. Furthermore, it increases in a time‐dependent manner; on the other hand, the TEAC values of **2** are slightly lower than those of **1** and **3**. These data suggest that the free radical scavenging capacity of the derivatives can be attributed to the high reactivity of the hydroxy groups on the aromatic rings as observed for other analogous systems.^19^ In the case of **2**, the hydroxy groups may be less available to react with the radical cation, probably due to the higher rigidity of the system relative to the analogous 6‐functionalized derivative. Similar behavior has also been reported for related cyclodextrin–deferiprone conjugates.^12^ However, the ability of **2** to scavenge free radicals is much higher than that of Trolox. This suggests that the CyD conjugates could be powerful antioxidants with activity similar to that of several polyphenols.

### Effect on α‐synuclein aggregation

The aggregation of proteins into toxic conformations plays a critical role in the development of various neurodegenerative disorders such as PD, Alzheimer's diseases (AD), and dementia with Lewy Bodies (DLB).^33^ DLB and PD are amongst a group of diseases referred to as α‐synucleinopathies, which are characterized by αSyn accumulation in cortical and subcortical regions.^34^ Therefore, compounds that can delay and/or prevent the aggregation process of αSyn could lead to a new therapeutic strategy for treating PD and other α‐synucleinopathies. The interaction of proteins with metals often plays a crucial role in the aggregation. In particular, copper(II) and iron(III) are the most effective ions in promoting αSyn aggregation.^35^

To investigate the influence of both CyD conjugates (**1** and **2**) on αSyn aggregation, we monitored the iron‐ and copper‐induced fibrillation of the protein in the absence and presence of **1**, **2**, and their parent compounds (**3** and β‐CyD) using a dynamic light scattering (DLS) technique. The effect of **3** on the metal‐induced αSyn aggregation was not determined because of the precipitation of its metal complexes under the assay conditions.

The hydrodynamic diameter of αSyn (mean diameter=6.4±0.3 nm) confirms the presence of the protein in a monomeric state, in keeping with data reported elsewhere.^36^, ^37^ Moreover, the size distribution of αSyn remains substantially unchanged after 24 hours under these conditions. The presence of Fe^3+^ and Cu^2+^ promotes aggregate formation.

DLS data indicate that both CD conjugates **1** and **2** efficiently inhibit metal‐induced αSyn aggregation, whereas unfunctionalized β‐CyD was unable to suppress the aggregation. In particular, **1** prevents αSyn aggregation at *t*=0 and shows only a small percentage of oligomers (5 %) with a mean diameter of 28 nm at *t*=24 h (Figure S20). Analogously, **2** is efficient in suppressing αSyn aggregation (Figure S21). Indeed, no aggregate is discernible at *t*=0 and *t*=24 h, only the size distribution (i.e., the polydispersity index) of the protein increased to a small extent. The behavior of these derivatives could be explained by the correlation between the capacity of inhibiting metal‐induced aggregation and their metal‐binding ability. As described above, **1**, **2**, and **3** form a 2:1 chelator/iron complex at physiological pH with high stability constant.^27^, ^28^ Compound **3** also has a moderate affinity for Cu^2+^ as demonstrated by the value of 18.8 for log β (CuL), and 23.9 for log β_2_ (CuL_2_) reported elsewhere.^38^ As observed for iron, we anticipate the conjugation with CyD does not significantly modify the copper‐binding ability of the chelators.^12^, ^31^ Moreover, we confirmed through UV/Vis and CD spectroscopy that both derivatives maintain the ability of **3** to complex copper ions. In particular, the absorption and CD bands of both CyD conjugates dramatically change upon addition of Cu^2+^ ions, indicating copper complexation (Figure S22). Overall, these systems can be used to gain control of iron and copper toxicity that is strictly implicated in protein aggregation.

### Effect on Niemann–Pick disease type C (NPC) cells

**Figure 7:**
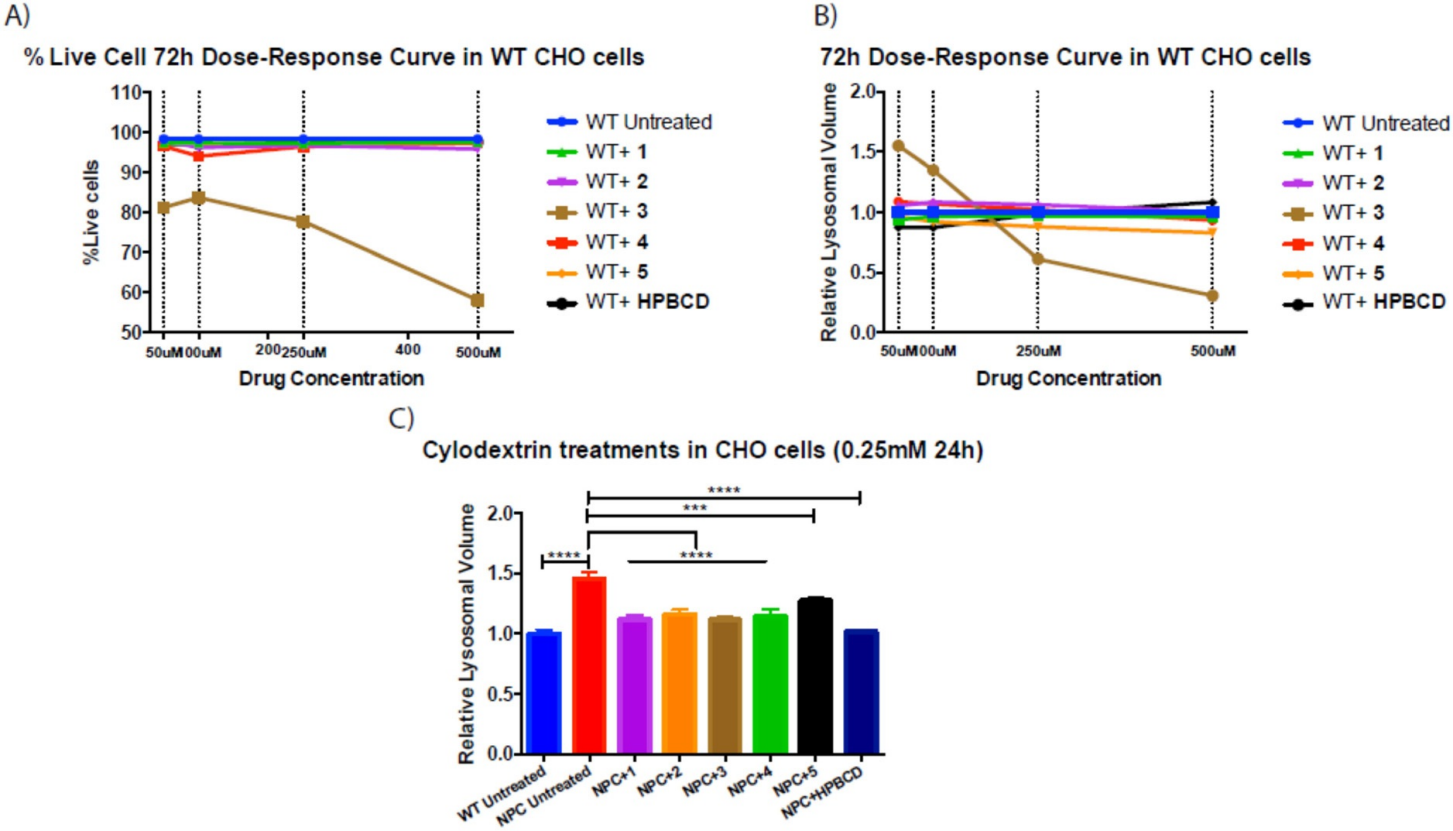
Effect of CyDs on NPC cells.

Because CyDs are applied in NPC and other neurodegenerative disorders, and CyD treatment has been shown to be beneficial and well‐tolerated in NPC cells, we tested **3** and its CyD conjugates **1** and **2** in NPC cells in comparison with HPBCD. For completeness, we also evaluated the known compound **4** (a dual action CyD‐Cu chelator)^12^ and the clioquinol‐like CyD analogue **5** (Figure S23) that belong to a class of CyD conjugates formerly proposed by us as multifunctional anti‐neurodegenerative agents. A dose–response study was performed on *Npc1* ^*+/+*^ (wild‐type) CHO cells using 72 hours of treatment to determine the cytotoxicity profiles. CyD compounds did not have any detrimental effects in terms of cell viability and did not affect relative lysosomal volume in the concentrations range tested (Figure 7). Conjugation with CyD significantly decreased the cytotoxicity of these compounds as observed in the case of other CyD conjugates.^39^ However, treatment with **3** over 72 hours significantly reduced viability at all doses tested relative to the untreated group and CyD‐treated cells in a dose‐dependent dependent manner (Figure 7 A). Also, **3** increased the acidic compartment volume of *Npc1* ^*+/+*^ CHO cells at low concentrations (55 % at 50 μm, 35 % at 100 μm) in wild‐type cells, while decreasing total acidic compartment volume at high concentrations (39 % at 250 μm, 69 % at 500 μm) (Figure 7 B).

These compounds were then tested on NPC1‐deficient CHO cells. *Npc1* ^*−/−*^ cells showed substantial increase in relative lysosomal volume (32 %) relative to *Npc1* ^*+/+*^ cells (*p<*0.0001) (Figure 7 C). Total acidic compartment volume decreased with **4** by 21.5 % (*p*<0.0001 normalized to *Npc1* ^*+/+*^ levels with *p*=0.0539), with **1** by 23.5 % (*p*<0.0001), **2** by 20.3 % (*p*<0.0001), **5** by 12.3 % (*p*<0.001), HPBCD by 30 % (*p*<0.0001 normalized to *Npc1* ^*+/+*^ levels with *p*=0.9995), and **3** by 23.5 % (*p*<0.0001 normalized to *Npc1* ^*+/+*^ levels with *p*=0.0534) (Figure 7 C).

A toxicity study (Table 3) in immortalized human liver carcinoma (HepG2) cells was carried out. This confirmed that the CyD–deferasirox conjugates **1** and **2** are substantially less toxic (or less antiproliferative) in cells than deferasirox alone (Figure S24). Solubility studies established that **1**–**3** had good solubility (>100 μm; Table S1).

**Table 3:** HepG2 toxicity study.

Compound	IC_50_ [μm]^[a]^
**1**	>100
**2**	>100
**3**	0.98^[b]^

[a] Values are the average of three replicates. [b] Estimated.

## Conclusions

Two novel CyD‐conjugated deferasirox analogues, **1** and **2**, were synthesized and thoroughly investigated. The derivatives have significant in vitro antioxidant capacity and can complex metal ions with high stability constants. Therefore, they can completely inhibit metal‐induced protein aggregation and the formation of amyloid fibrils. These features of **1** and **2** are highly desirable considering the critical involvement of metal ions in protein misfolding and aggregation, which are hallmarks of several neurodegenerative disorders.

Furthermore, the cyclodextrin moiety renders these systems less toxic than deferasirox, which is well known for its side effects when administrated to treat iron‐overload diseases. The most important advantages of both **1** and **2** are their good solubility in water and the possibility of forming inclusion complexes to include endogenous compounds, such as cholesterol, correcting lysosomal volume in NPC cells. Compared with the other CyD conjugates previously studied by us,^12^, ^13^ **1** and **2** are the first examples of nontoxic conjugates of a drug on the market that we have studied in a model of NPC. Overall, the conjugates hold promise as multitargeted therapies in the treatment of disorders of metal dyshomeostasis and lipid imbalances.

## Experimental Section

**Chemicals**: 6^A^‐Amino‐6^A^‐deoxy‐β‐cyclodextrin was synthesized by a microwave‐assisted procedure starting from the corresponding 6‐tosylate derivative as reported elsewhere,^40^ whereas 3^A^‐amino‐3^A^‐deoxy‐2^A^(*S*),3^A^(*S*)‐β‐cyclodextrin was obtained from TCI. Starting materials were purchased from Molekula (UK) or Cyclolab (Hungary), and human αSyn was purchased from Sigma–Aldrich. Cu^2+^ stock solutions were prepared by dissolving the corresponding perchlorate in water and titrating the resulting solutions with standardized EDTA by using murexide. Ferric ion solutions were prepared by dissolving FeCl_3_⋅6 H_2_O in 0.01 m hydrochloric acid to prevent hydrolysis. Iron solution titer was spectrophotometrically determined on the Fe–desferal complex at 428 nm. For clarity, the sugar units in β‐CyD derivatives are labeled A–G counter‐clockwise starting from the modified ring (denoted as A) and viewing from the upper rim (Figure S25).

**Synthesis of 4‐[3,5‐bis(2‐hydroxyphenyl)‐1*H*‐1,2,4‐triazol‐1‐yl]‐*N*‐[6^A^‐amino‐6^A^‐deoxy‐β‐cyclodextrin]benzamide (1)**: HOBt (24 mg, 0.175 mmol), DCC (36 mg, 0.175 mmol) and triethylamine (50 μL, 0.53 mmol) were added to a solution of **3** (65.5 mg, 0.175 mmol) in dry DMF (5 mL). After 20 min, 6^A^‐amino‐6^A^‐deoxy‐β‐cyclodextrin (205 mg, 0.175 mmol) was added. The reaction was stirred at room temperature under argon for 48 h. The solvent was evaporated to dryness in vacuo. The crude product was then triturated with diethyl ether (3×3 mL) followed by a cold acetone trituration (3×4 mL) and hot acetone trituration (3×3 mL), and the crude solids were purified by reversed‐phase chromatography using a 30 g Biotage KP‐RC‐18 column (eluent: H_2_O→CH_3_OH) to give the title product as a white solid. For numbering of **1** refer to Figure 1. Yield: 64 % (168 mg, 0.11 mmol); TLC: *R*_f_=0.28 (*i*PrOH/EtOAc/H_2_O/NH_3_ 4:3:2:1); ^1^H NMR (500 MHz, D_2_O): *δ*=7.95 (d, *J*_*13,14*_=7.7 Hz, 1 H, H‐13), 7.48 (t, *J*=7.8 Hz, 1 H, H‐15), 7.39 (m, 1 H, H‐21), 7.31 (d, *J*_*8,7*_=*J*_*10,11*_=8.0 Hz, 2 H, H‐8 and H‐10), 7.26 (d, *J*_*23,22*_=7.5 Hz, 1 H, H‐23), 7.09 (d, *J*_*16,15*_=8.2 Hz, 1 H, H‐16), 7.04 (m, 4 H, H‐7, H‐11, H‐14 and H‐22), 6.79 (d, *J*_*20,21*_=7.8 Hz, 1 H, H‐20), 5.02 (d, *J*_*1G,2G*_=3.6 Hz, 1 H, H‐1G of CyD), 4.96 (d, *J*=3.5 Hz, 3 H, Hs‐1 of CyD), 4.92 (d, *J*=3.6 Hz, 1 H, H‐1A of CyD), 4.82 (d, *J*=3.6 Hz, 2 H, Hs‐1 of CyD), 4.05 (d, *J*_*6A,6A‘*_=13.7 Hz, 1 H, H‐6A of CyD), 4.01–3.16 (m, 39 H, Hs‐2, Hs‐3, Hs‐4 and Hs‐5 of CyD), 3.16–2.96 ppm (m, 2 H, H‐6A and H‐6X of CyD); ^13^C NMR (126 MHz, D_2_O): *δ*=166.0 (C=O), 160.6 (C3), 155.9 (C17), 154.9 (C19), 152.5 (C5), 139.8 (C6), 133.1 (C21), 133.4 (C9), 131.9 (C15), 130.7 (C23), 127.8 (C8, C10), 126.9 (C13), 122.1 (C7 and C11), 120.0 (C22 and C14), 116.8 (C16), 116.4 (C20), 112.9 (C12 and C18), 102.3 (C1G of CyD), 101.8 (Cs‐1 of CyD), 101.4 (C1A of CyD), 84.0 (C4A of CyD), 80.3 (Cs‐4 of CyD), 73.1–71.6 (Cs‐2, Cs‐3 and Cs‐5 of CyD), 59.6 (Cs‐6 of CyD), 41.1 ppm (C6A of CyD); CD (H_2_O) *λ* nm^−1^ (Δ*ϵ*): 211 (+1.4), 233 (−3.6), 275 (−1.7), 315 (+0.6); ESI‐MS: *m*/*z*=1490.45 [*M*+H]^+^; Analytical 8 min run, H_2_O/acetonitrile (95:5 to 5:95); *t*_R_=2.59 min; purity=100 % (Figures S26–28).

**Synthesis of 4‐[3,5‐bis(2‐hydroxyphenyl)‐1*H*‐1,2,4‐triazol‐1‐yl]‐*N*‐[3^A^‐deoxy‐3^A^‐amino‐β‐cyclodextrin]benzamide (2)**: HOBt (24 mg, 0.175 mmol), DCC (36 mg, 0.175 mmol) were added to a solution of **3** (65.5 mg, 0.175 mmol) in dry DMF (5 mL). After 20 min, 3^A^‐amino‐3^A^‐deoxy‐β‐cyclodextrin (199 mg, 0.175 mmol) was added. The reaction was stirred at room temperature under argon for 48 h. The solvent was evaporated to dryness in vacuo. The crude product was then triturated with diethyl ether (3×3 mL), followed by a cold acetone trituration (3×4 mL) and hot acetone trituration (3×3 mL), and the crude solids were purified by reversed‐phase chromatography using a 30 g Biotage KP‐RC‐18 column (eluent: H_2_O→CH_3_OH) to give the title product as a white solid. Refer to Figure 1 for the numbering of compound **2**. Yield: 57 % (149 mg, 0.10 mmol); TLC: *R*_f_=0.43 (*i*PrOH/EtOAc/H_2_O/NH_3_ 4:3:2:1); ^1^H NMR (500 MHz, D_2_O): *δ*=7.94 (d, *J*=7.7 Hz, 1 H, H‐13), 7.80 (bs, 2 H, H‐8 and H‐10), 7.64–7.26 (m, 5 H, H‐7, H‐11, H‐15, H‐21 and H‐23), 7.14–6.95 (m, 3 H, H‐14, H‐16 and H‐22), 6.71 (s, 1 H, H‐20), 5.09–4.99 (m, 2 H, Hs‐1 of CyD), 4.97 (d, *J*=3.4 Hz, 1 H, H‐1 of CyD), 4.85 (s, 2 H, Hs‐1 of CyD), 4.81 (s, 1 H, H‐1 of CyD), 4.59 (bs, 1 H, H‐1A of CyD), 4.46–2.78 (m, 42 H, Hs‐2, Hs‐3, Hs‐4, Hs‐5 and Hs‐6 of CyD); ^13^C NMR (126 MHz, [D_6_]DMSO): *δ*=165.4 (C=O), 160.2 (C3), 156.8 (C17), 155.7 (C19), 152.4 (C5), 140.2 (C6), 134.6 (C9), 132.9 (C21), 131.8 (C15), 131.4 (C23), 128.8 (C8, C10), 127.1 (C13), 123.6 (C7 and C11), 120.0 (C22 and C14), 117.5 (C16), 116.6 (C20), 114.9 (C12), 114.1 (C18), 104.6 (C1A of CyD), 102.4–102.0 (Cs‐1 of CyD), 82.4–81.0 (Cs‐4 of CyD), 79.9 (C5A of CyD), 73.6–71.2 (Cs‐2, Cs‐3 and Cs‐5 of CyD), 60.7–60.0 (Cs‐6 of CyD), 51.7 (C3A); ESI‐MS: *m*/*z*=764.2 [*P*+H+K]^2+^, 1490.45 [*M*+H]; CD (H_2_O) *λ* nm^−1^ (Δ*ϵ*): 222 (+9.4), 233 (−10.2), 275 (−11.2), 315 (+4.5); Analytical 8 min run, H_2_O/acetonitrile (95:5 to 5:95). *t*_R_=2.45 min; purity=99 %.

**Synthesis of 6^A^‐deoxy‐6^A^‐[{(5‐bromo‐8‐hydroxyquinolyl)‐2‐carboxyl}amino]‐β‐cyclodextrin (5)**: HOBt (22 mg, 0.16 mmol), DCC (33 mg, 0.16 mmol) and 5‐bromo‐8‐hydroxyquinoline‐2‐carboxylic acid (Scheme S1; 43 mg, 0.16 mmol) were dissolved in anhydrous DMF (4.0 mL) under inert conditions. To this solution NEt_3_ (45 μL, 0.32 mmol) was added followed by 6‐monodeoxy‐6‐monoamino‐β‐cyclodextrin (187 mg, 0.16 mmol), added portion‐wise over 30 min at room temperature with vigorous stirring. The reaction mixture was stirred overnight under nitrogen. After 12 h, DCC (33 mg, 0.16 mmol), HOBt (22 mg, 0.16 mmol) and NEt_3_ (22.5 μL, 1.0 equiv) were further added, and the reaction was heated at 50 °C for 5 days. Upon completion the reaction mixture was concentrated to dryness before trituration of the crude solid using diethyl ether (3×5 mL) was undertaken. The ether was decanted, and the resulting solid was triturated using hot acetone (6×6 mL) and the solid product was dried by desiccation. The material was purified using reversed‐phase chromatography (30 g C18 Biotage Column) using liquid loading in H_2_O/DMSO (1:2, 5 mL). The purification was run using a solvent system of H_2_O/MeOH (100:0–0:100) yielding the pure final product **5** (124 mg, 56 % yield, *R*_f_=0.5 (EtOAc/*i*PrOH/H_2_O/NH_3_ aq. (4:3:2:1)); ^1^H NMR (600 MHz, [D_6_]DMSO): *δ*=10.22 (s, 1 H, NH), 9.53 (dd, *J*=7.5, 4.4 Hz, 1 H, OH of HQ), 8.57 (d, *J*=8.7 Hz, 1 H, H‐3 of HQ), 8.24 (d, *J*=8.7 Hz, 1 H, H‐4 of HQ), 7.86 (d, *J*=8.3 Hz, 1 H, H‐6 of HQ), 7.10 (d, *J*=8.3 Hz, 1 H, H‐7 of HQ), 5.94 (d, *J*=6.0 Hz, 1 H, OH of CyD), 5.86 (dd, *J*=16.6, 6.4 Hz, 2 H, OH of CyD), 5.75 (d, *J*=11.4 Hz, 3 H, OH of CyD), 5.72–5.65 (m, 6 H, OH of CyD), 5.63 (s, 1 H, OH of CyD), 5.51 (d, *J*=7.5 Hz, 1 H, OH of CyD), 4.98 (d, *J*=3.7 Hz, 1 H, H‐1 of CyD), 4.84 (m, 3 H, H‐1 of CyD), 4.81 (d, *J*=3.5 Hz, 1 H, H‐1 of CyD), 4.76 (d, *J*=3.6 Hz, 1 H, H‐1 of CyD), 4.65 (d, *J*=3.5 Hz, 1 H, H‐1 of CyD), 4.53 (s, 1 H), 4.50 (s, 1 H), 4.45 (s, 2 H), 4.25 (s, 1 H), 4.00–3.94 (m, 1 H), 3.86 (dd, *J*=12.3, 4.1 Hz, 1 H), 3.81 (d, *J*=11.2 Hz, 1 H), 3.72 (d, *J*=13.2 Hz, 1 H), 3.69 (s, 1 H), 3.72–3.66 (m, 1 H), 3.68–3.60 (m, 8 H), 3.63–3.59 (m, 2 H), 3.57 (d, *J*=11.4 Hz, 2 H), 3.56–3.49 (m, 4 H), 3.42–3.33 (m, 8 H), 3.17 (t, *J*=5.9 Hz, 1 H), 2.56–2.50 ppm (m, 1 H); ^13^C NMR (151 MHz, [D_6_]DMSO): *δ*=163.4 (C=O), 154.4 (C8 of HQ), 148.6 (C2 of HQ), 137.6 (C4 of HQ), 137.2 (C9 of HQ), 133.1 (C6 of HQ), 128.4 (C10 of HQ), 120.8 (C3 of HQ), 113.0 (C7 of HQ), 108.7 (C5 of HQ), 102.7 (C1 of CyD), 102.7 (C1 of CyD), 102.6 (C1 of CyD), 102.3 (C1 of CyD), 102.1 (C1 of CyD), 101.5 (C1 of CyD), 85.03 (C4A of CyD), 82.3–80.9 (Cs‐4 of CyD), 74.2–70.5 (Cs‐3, Cs‐5, Cs‐2 of CyD), 60.5 (C6 of CyD), 60.3 (C6 of CyD), 60.2 (C6 of CyD), 60.1 (C6 of CyD), 59.6 (C6 of CyD), 58.9 (C6 of CyD), 40.6 ppm (C6A of CyD). HRMS: *m*/*z*=1405.3227 [*M*+Na]^+^. Analytical 8 min run, H_2_O/acetonitrile (70:30 to 5:95); *t*_R_=1.84 min; purity=100 %.

**NMR spectroscopy and mass spectrometry**: ^1^H and ^13^C NMR spectra were recorded at 25 °C with a Varian UNITY PLUS‐500 spectrometer at 499.9 and 126 MHz, respectively. The NMR spectra were obtained by using standard pulse programs from the Varian library. The 2D experiments (COSY, TOCSY, gHSQCAD, gHMBC, ROESY) were acquired by using 1000 data points, 256 increments, and a relaxation delay of 1.2 s. The spectra were referred to as the solvent signal.

**UV/Vis and CD spectroscopy**: UV/Vis spectra were recorded on an Agilent 8452A diode array spectrophotometer. Circular dichroism measurements were performed on a JASCO spectropolarimeter (model J‐1500).

**Iron stability constant determination**: The automated titration system consisted of an auto‐burette (Metrohm Dosimat 765 liter mL syringe), a Mettler Toledo MP230 pH meter with SENTEK pH electrode (P11), and a d‐type 8453 UV/visible spectrophotometer with a Hellem quartz flow cuvette being circulated through by a Gilson Mini‐plus #3 pump operated at a flow rate of 20 mL min^−1^. A potassium chloride electrolyte solution (0.1 m) was used to maintain ionic strength. The temperature of the test solutions was maintained in a thermostatic jacketed titration vessel at 25±0.1 °C using a Fisherbrand Isotemp water bath. The pH electrodes were calibrated using GLEE^41^ with data obtained by titrating a volumetric standard HCl (0.1 m) in KCl (0.1 m) with KOH (0.1 m) under an argon gas atmosphere in the vessel. The solution under investigation was stirred vigorously during the experiment. For p*K*_a_ determinations, a cuvette path length of 10 mm was used, whereas for metal stability constant determinations, a cuvette path length of 50 mm was used. All instruments were interfaced to a computer and controlled by an in‐house program. An automated titration adopted the following strategy: the pH of a solution was increased by 0.1 pH unit by the addition of potassium hydroxide solution (0.1 m) from the auto‐burette. The pH readings were judged to be stable if their values varied by less than 0.01 pH unit after a set incubation period. For p*K*_a_ determinations, an incubation period of 1.5 min was adopted; for metal stability constant determinations, an incubation period of 3 min was adopted. The cycle was repeated until the defined end‐point pH value was achieved. Titrations were carried out in DMSO/H_2_O solution at a molar ratio of 0.2:1 due to the solubility issue of the three analogues and/or corresponding iron complexes. Under these conditions, the pH meter readings are shifted relative to aqueous solution. All titration data were analyzed with HypSpec2014 software^42^, ^43^ (http://www.hyperquad.co.uk/). The speciation plot was calculated with the HYSS program.^44^ Analytical‐grade reagents were used in the preparation of all solutions.

**DLS measurements**: Dynamic light scattering (DLS) measurements were carried out using a Zetasizer Nano ZS (Malvern Instruments, UK) equipped for backscattering at 173° with a 633 nm He–Ne laser. Each DLS measurement was run using automated optimal measurement times, and laser attenuation settings according to the literature.^31^

**Trolox equivalent antioxidant capacity assay**: In vitro antioxidant assays were performed by 2,2′‐azinobis(3‐ethylbenzothiazoline‐6‐sulfonic acid) diammonium salt (ABTS^•+^) radical cation decolorization assay using 6‐hydroxy‐2,5,7,8‐tetramethylchroman‐2‐carboxylic acid (Trolox) as reported elsewhere.^45^ In brief, the radical cation ABTS^•+^ was generated by a reaction between ABTS (7 mm) and persulfate (2.45 mm) in water for 16 h (dark, room temperature). This radical solution was diluted in phosphate buffer (10 mm, pH 7.4) and combined with various concentrations of the sample antioxidants (**1**, **2**, and **3**). All samples were diluted approximately to provide 20–80 % inhibition of the blank absorbance. The absorbance values were measured for 6 min. Solution absorbance was plotted versus test compound concentration; each resultant slope was normalized to that obtained for Trolox to give the Trolox‐equivalence (TEAC) value for each time point (1, 3, 6 min). All measurements were performed in triplicate.

**Anti‐aggregation assay**: αSyn solutions (0.5 mg mL^−1^) were buffered at pH 6.6 and 7.4 (MOPS, 20 mm). Iron‐ and copper‐mediated aggregation of αSyn was studied using suitable FeCl_3_ and Cu(ClO_4_)_2_ solutions in order to have a final concentration of 265 μm in cuvette as reported elsewhere.^37^ CyD derivatives were added in an equimolar amount to the copper ions. Each DLS measurement was run by using automated, optimal measurement times, and laser attenuation settings.

**Cell lines**: Wild‐type and *Npc1* ^*−/−*^ null CHO cells were used, as per the report by Higaki et al.^46^

**LysoTracker Green and propidium iodide staining**: In vitro FACS experiments were carried out to measure acidic compartment volumes according to a published method.^47^ Live cells were stained with LysoTracker™ Green DND‐26 (Thermo Fisher‐L7526) at 250 nm for 10 min in PBS at RT, centrifuged at 1200×*g*, 10 min and cells resuspended in FACS buffer (PBS, 1 % BSA, 0.1 % NaN_3_). Cells were stained with propidium iodide (20 nm; Invitrogen P3566) immediately before analysis on the FACS machine for dead cell separation. FACS analyses were performed with 10 000 recorded cells by using FACS Canto with BD software.

## Conflict of interest

*The authors declare no conflict of interest*.

## Supporting information

As a service to our authors and readers, this journal provides supporting information supplied by the authors. Such materials are peer reviewed and may be re‐organized for online delivery, but are not copy‐edited or typeset. Technical support issues arising from supporting information (other than missing files) should be addressed to the authors.

Supplementary
