# Supplementary material for: Synthesis and Study of Multifunctional Cyclodextrin–Deferasirox Hybrids
Source: ChemMedChem. 2019 Jun 24;14(16):1484–92. doi: 10.1002/cmdc.201900334 (PMC6771688; doi:10.1002/cmdc.201900334)
Supplement: Supplementary file 1 — Supplementary [file CMDC-14-1484-s001.pdf]

CHEMMEDCHEM
Chemistry & Drug Discovery

Supporting Information

Synthesis and Study of Multifunctional Cyclodextrin–Deferasirox Hybrids

Jose Miguel Gascon,[a] Valentina Oliveri,*[a, b] Andrew McGown,[a] Ecem Kaya,[c] Yu-Lin Chen,[d] Carol Austin,[e] Martin Walker,[e] Frances M. Platt,*[c] Graziella Vecchio,*[b] and John Spencer*[a]

cmdc_201900334_sm_miscellaneous_information.pdf

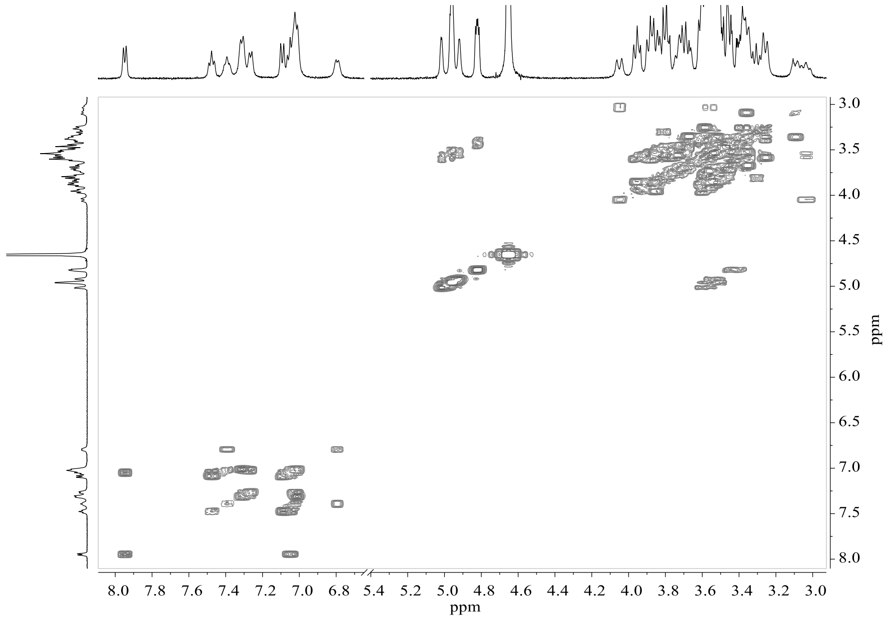

Figure S1. COSY spectrum of 1 in D_2O at 500 MHz.

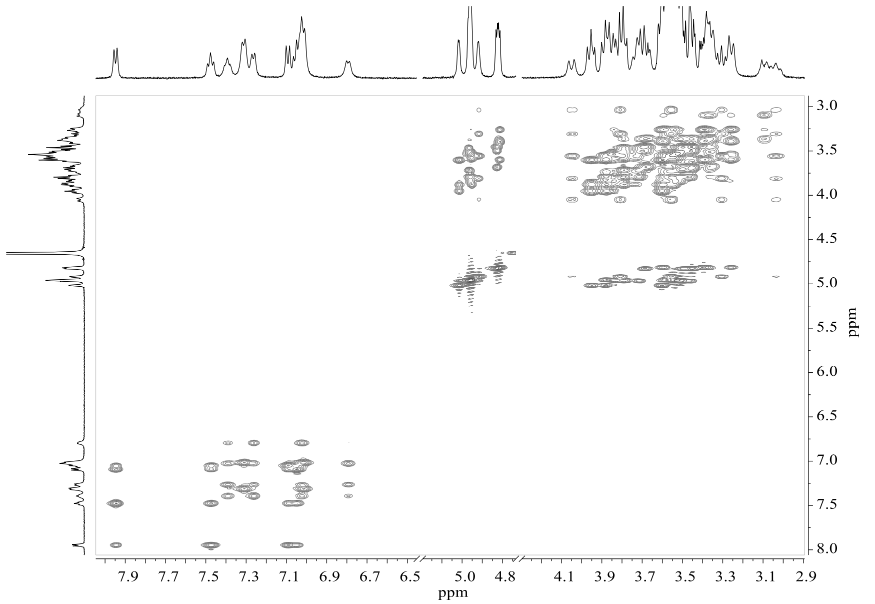

Figure S2. TOCSY spectrum of 1 in D_2O at 500 MHz.

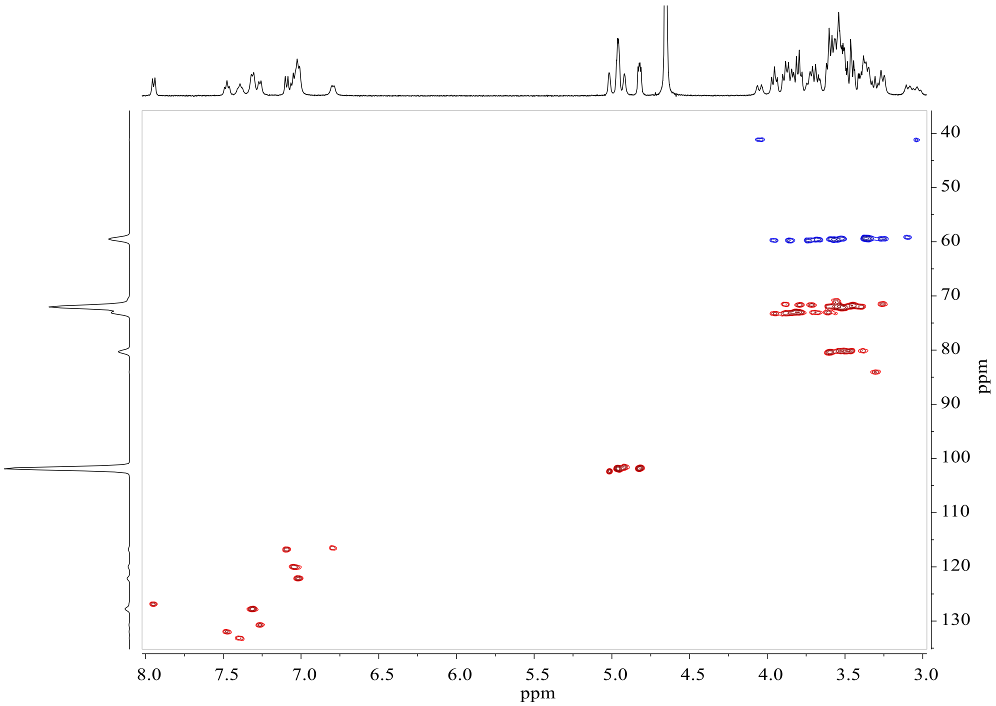

Figure S3. HSQCAD spectrum of 1 in D_2O.

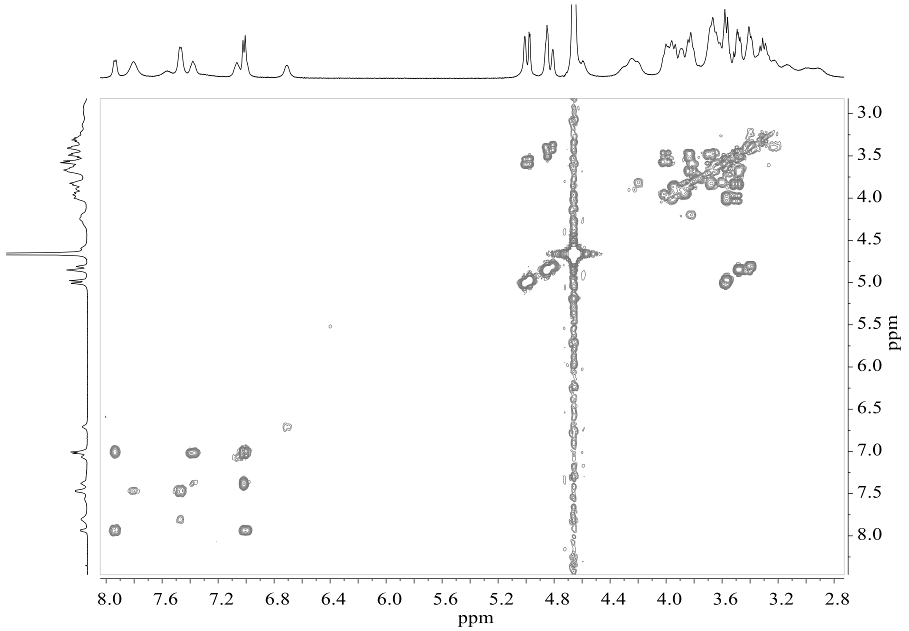

Figure S4. COSY spectrum of 2 in D_2O at 500 MHz.

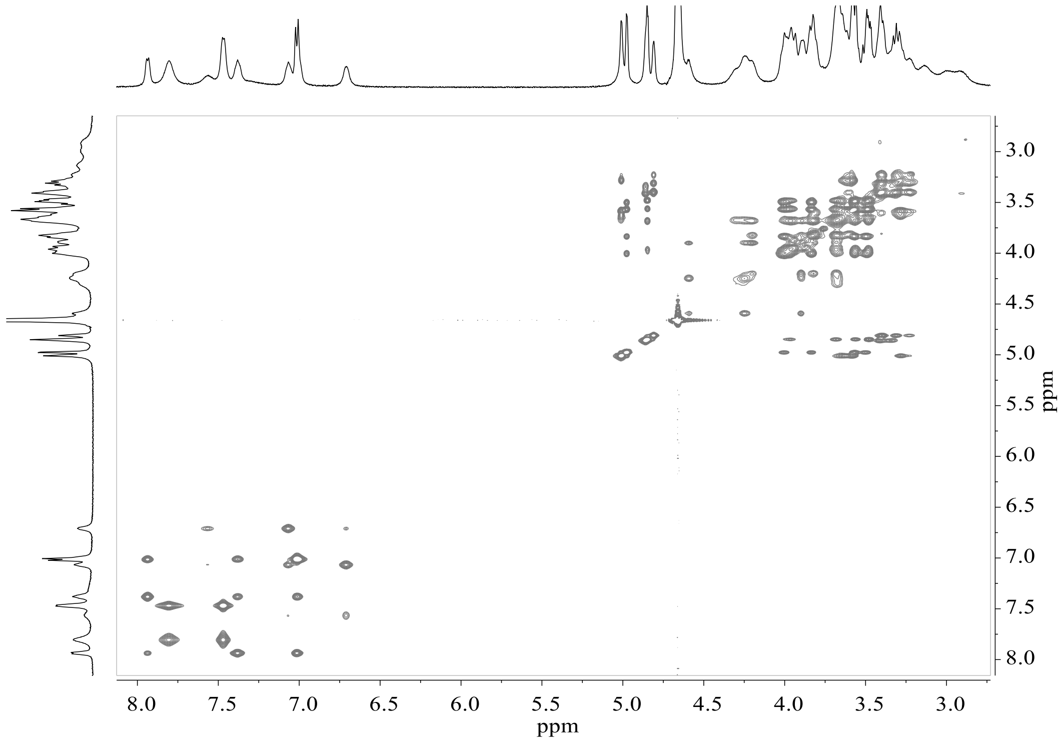

Figure S5. TOCSY of 2 in D_2O at 500 MHz.

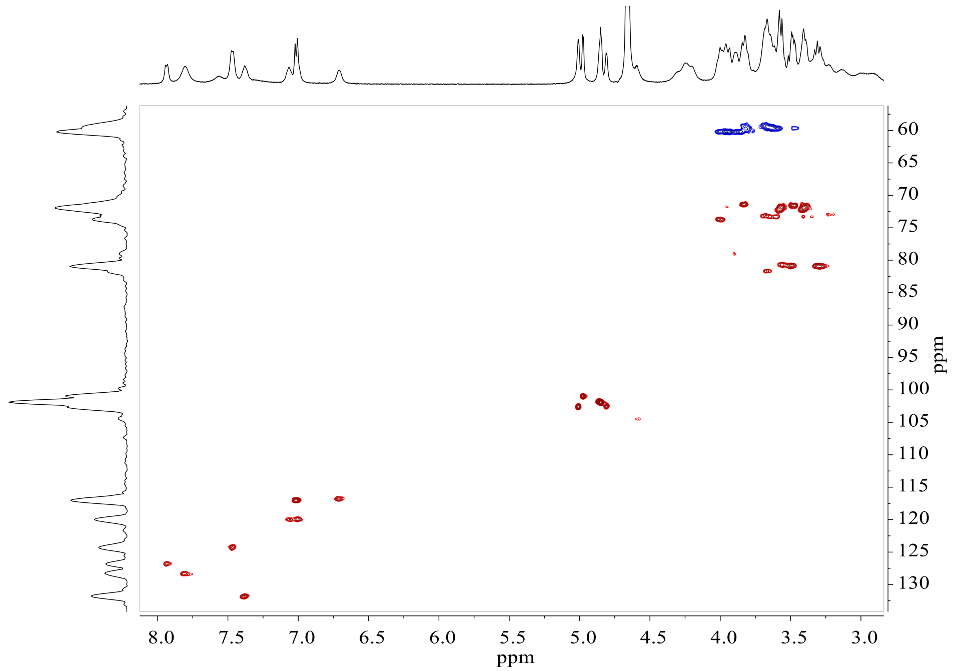

Figure S6. HSQCAD of 2 in D_2O.

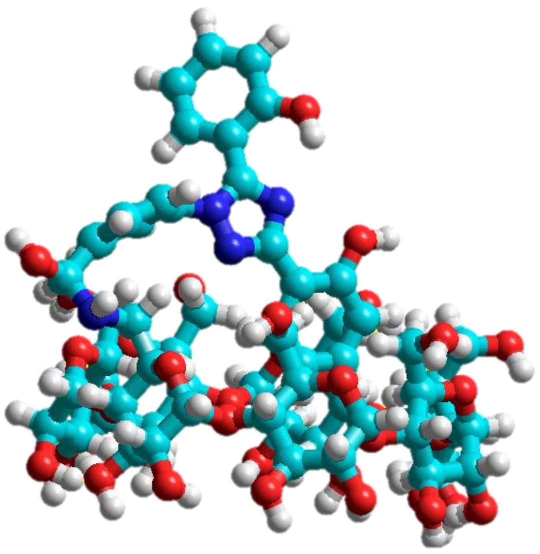

Figure S7. Hypothesized 3D molecular structure of 1 in water at pH 6.8.

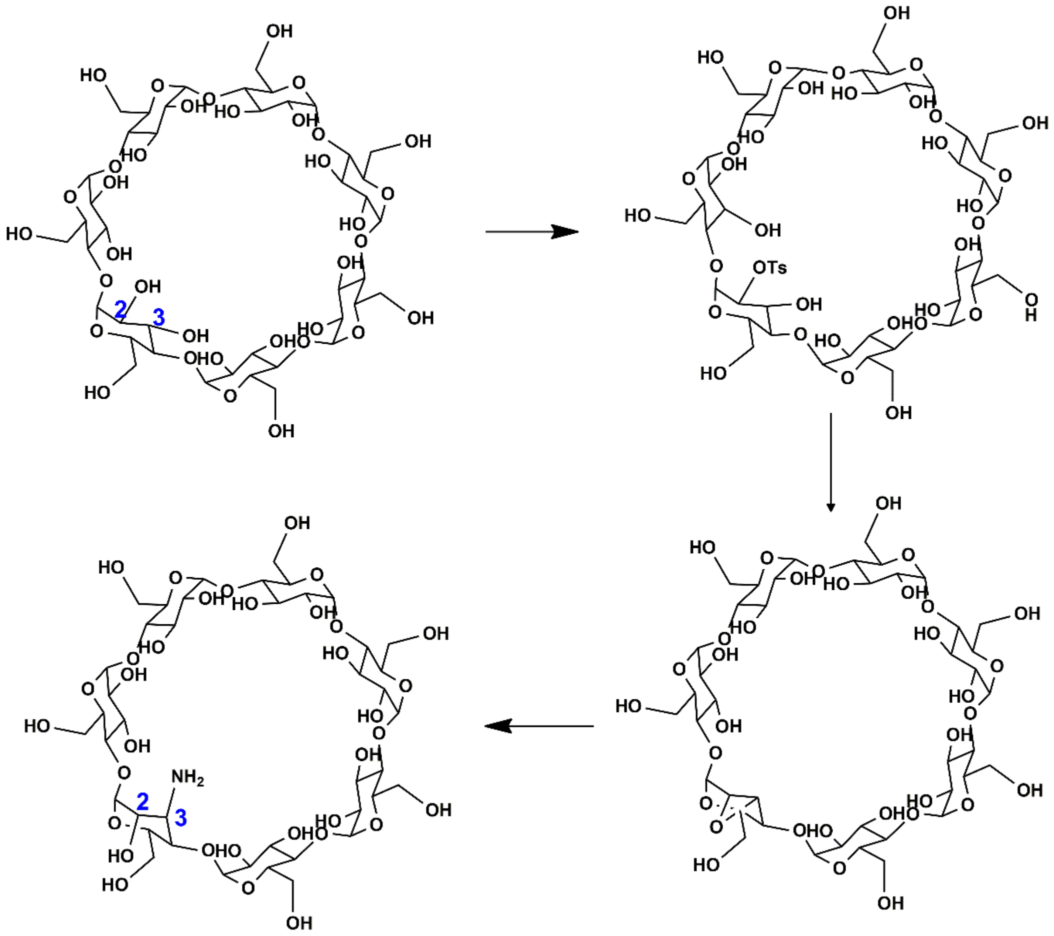

Figure S8. Synthesis scheme of 3^A^-deoxy-3^A^-amino-β-cyclodextrin.

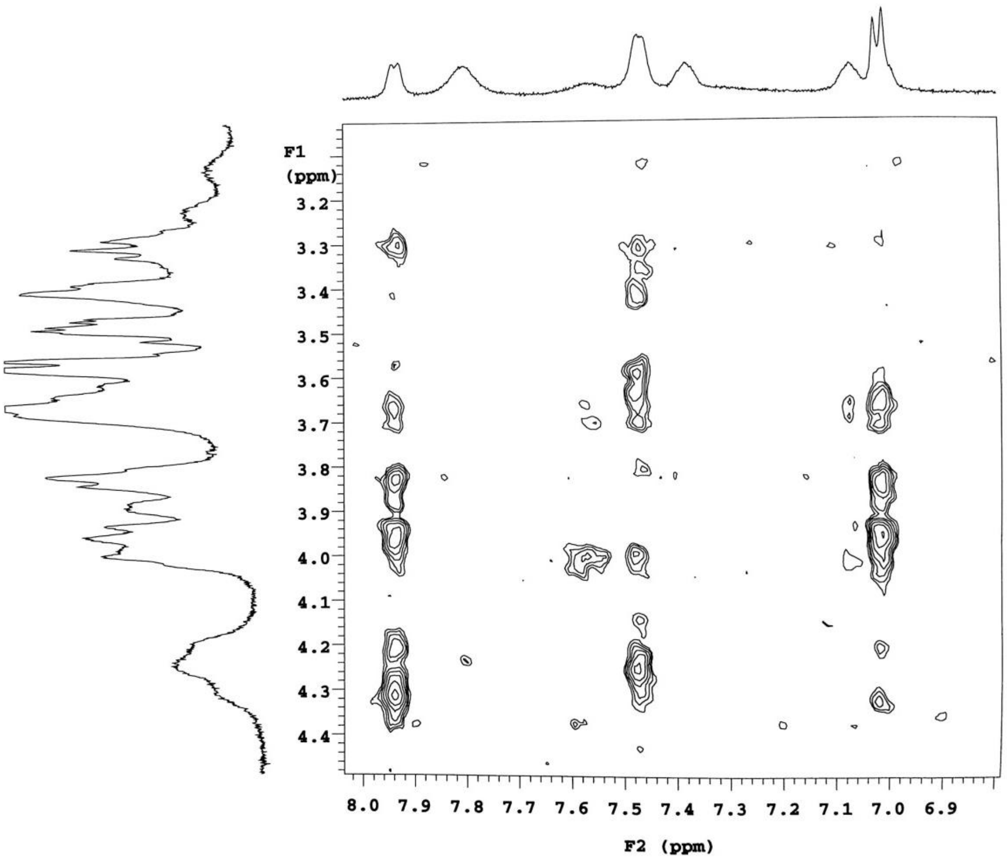

Figure S9. ROESY spectrum of 2 in D_2O at 500 MHz.

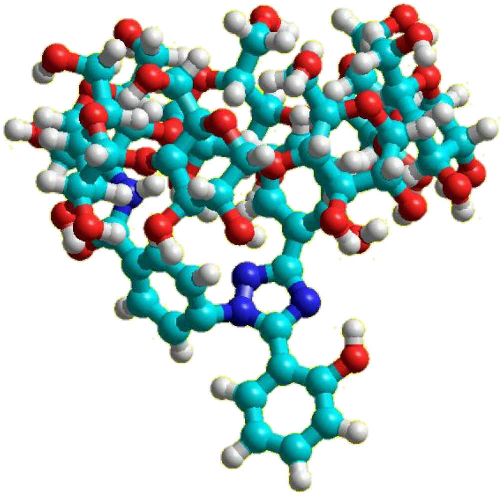

Figure S10. Hypothesized 3D molecular structure of 2 in water at pH 6.8.

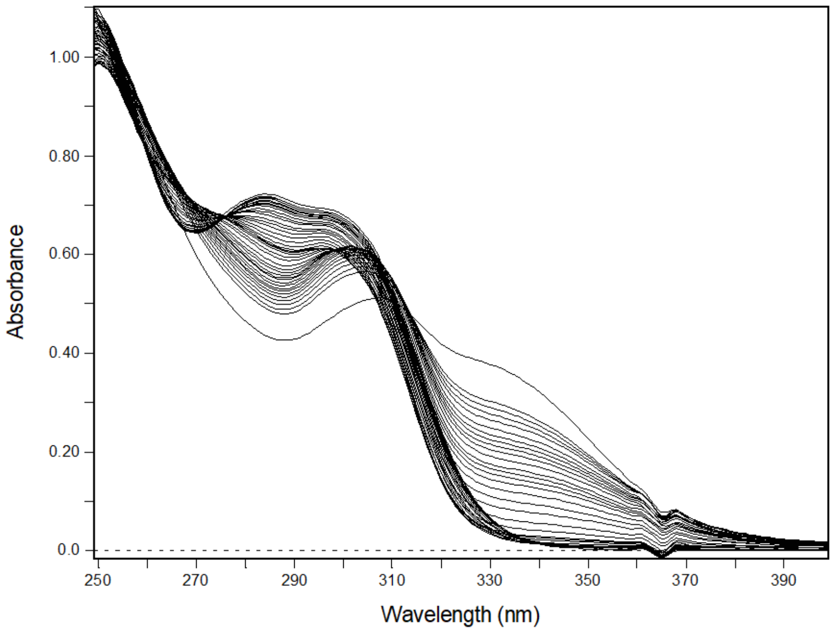

Figure S8. UV spectra of 3. [3] = 43.2 µM (started in 15.450 mL of the solution with molar ratio of DMSO : H_2O being 0.2 : 1), pH was changed from 3.06 to 12.45 by the addition of 0.1 M KOH at 25 °C.

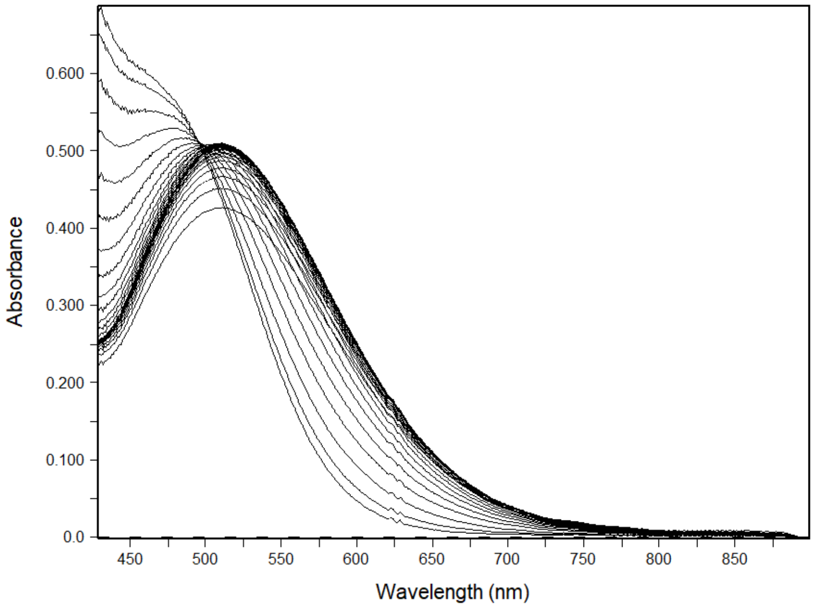

Figure S9. UV spectra of 3 in the presence of iron. [3] = 114 µM, [Fe^{3+}] = 38.0 µM (started in 15.226 mL of the solution with molar ratio of DMSO : H_2O being 0.2 : 1), pH was changed from 2.55 to pH 7.20 by the addition of 0.1 M KOH at 25 °C.

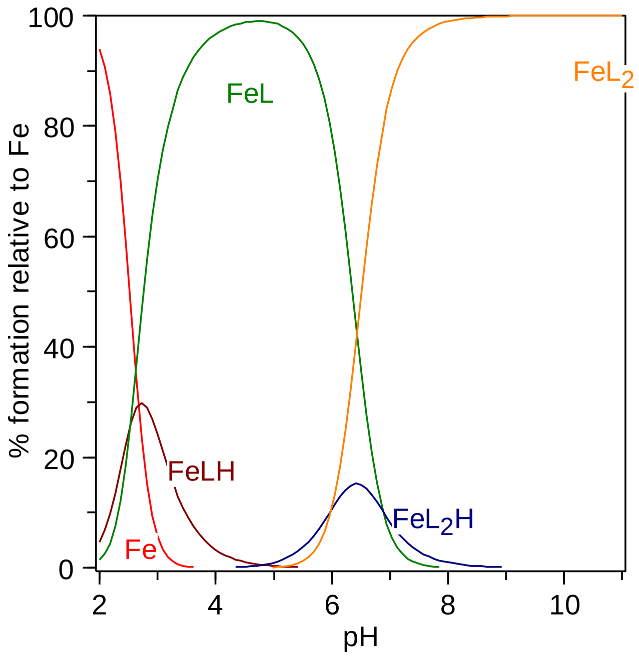

Figure S13. Distribution diagrams for the complex species of 3 ([Fe]total: 1.000E-06M [L]total: 1.000E-05M). [2]

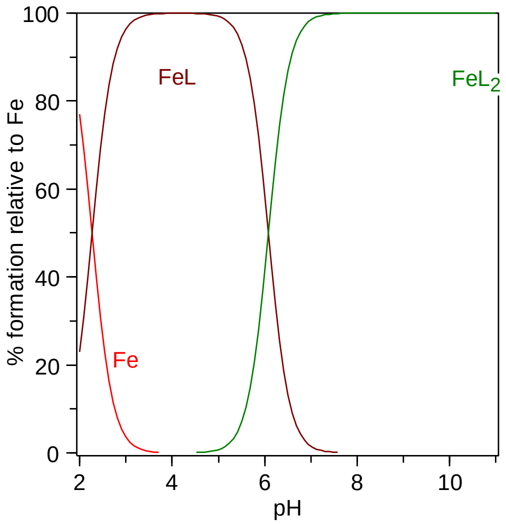

Figure S10. Distribution diagrams for the complex species of 1 ([Fe]total: 1.000E-06M [L]total: 1.000E-05M)

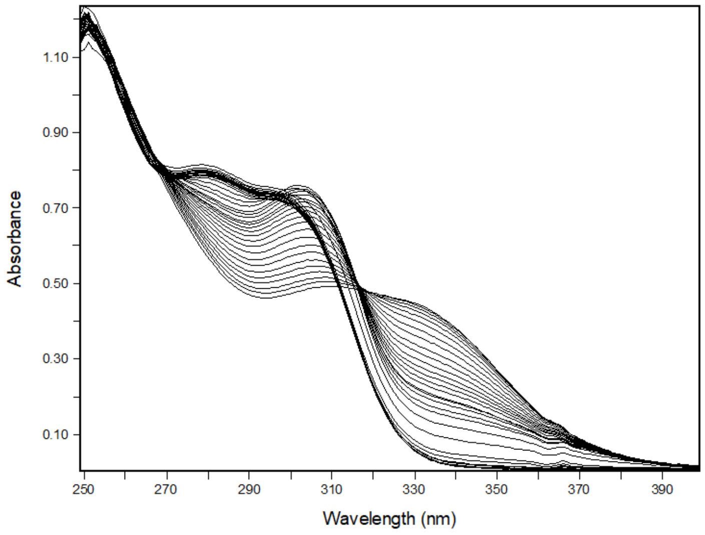

Figure S11. UV spectra of 2. [2] = 55.6 μM (started in 15.660 mL of the solution with molar ratio of DMSO : H_2O being 0.2 : 1), pH was changed from 2.89 to 12.99 by the addition of 0.1 M KOH at 25 °C.

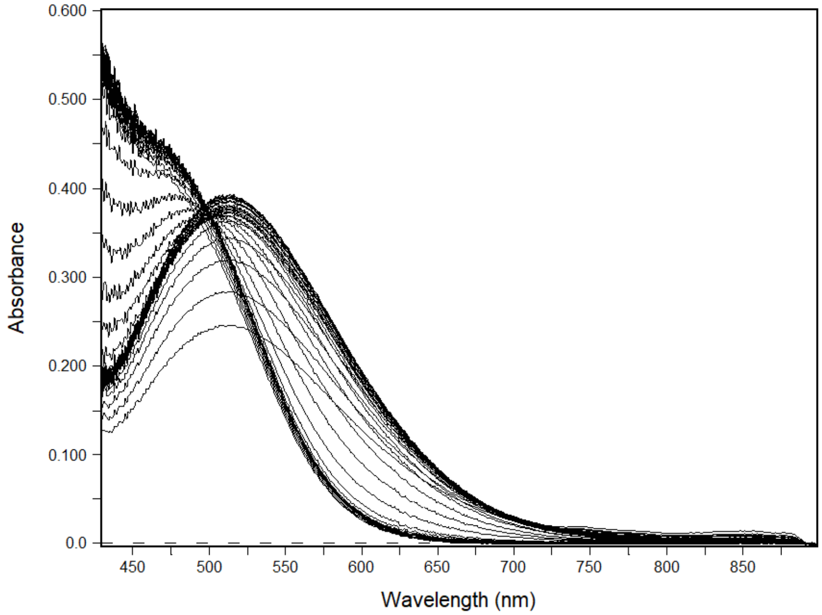

Figure S12. UV spectra of 2 in the presence of iron. [2] = 95.0 μM, [Fe^{3+}] = 35.6 μM (started in 15.260 mL of the solution with molar ratio of DMSO : H_2O being 0.2 : 1), pH was changed from 2.17 to pH 11.54 by the addition of 0.1 M KOH at 25 °C.

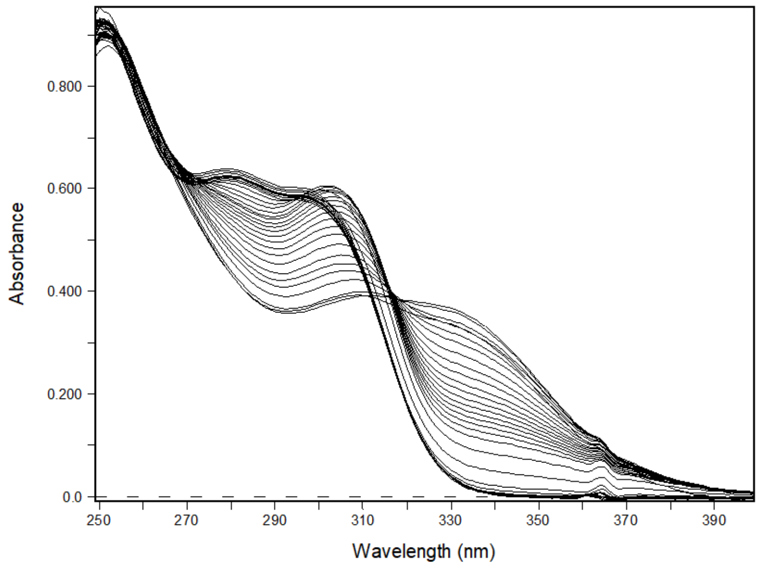

Figure S13. UV spectra of 1. [1] = 45.4 µM (started in 15.660 mL of the solution with molar ratio of DMSO : H_2O being 0.2 : 1), pH was changed from 2.94 to 12.99 by the addition of 0.1 M KOH at 25 °C.

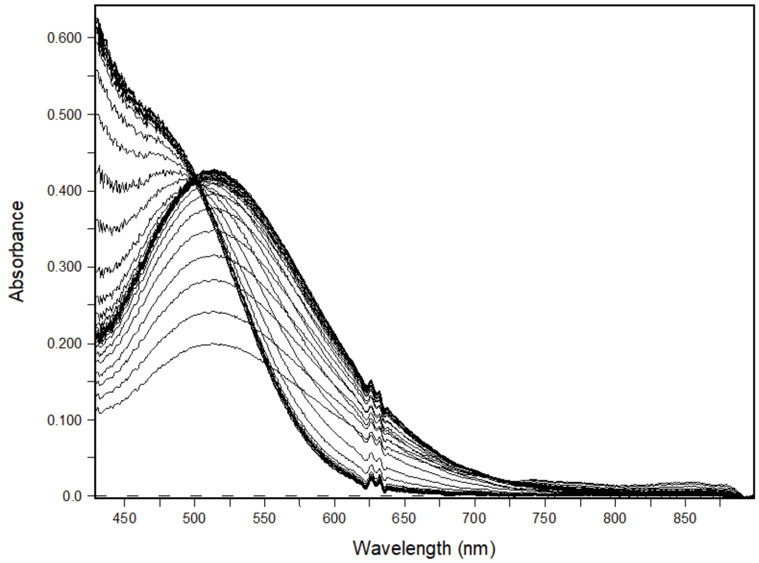

Figure S18. UV spectra of 1 in the presence of iron. [1] = 77.7 µM, [Fe^{3+}] = 35.6 µM (started in 15.260 mL of the solution with molar ratio of DMSO : H_2O being 0.2 : 1), pH was changed from 2.02 to pH 11.64 by the addition of 0.1 M KOH at 25 °C.

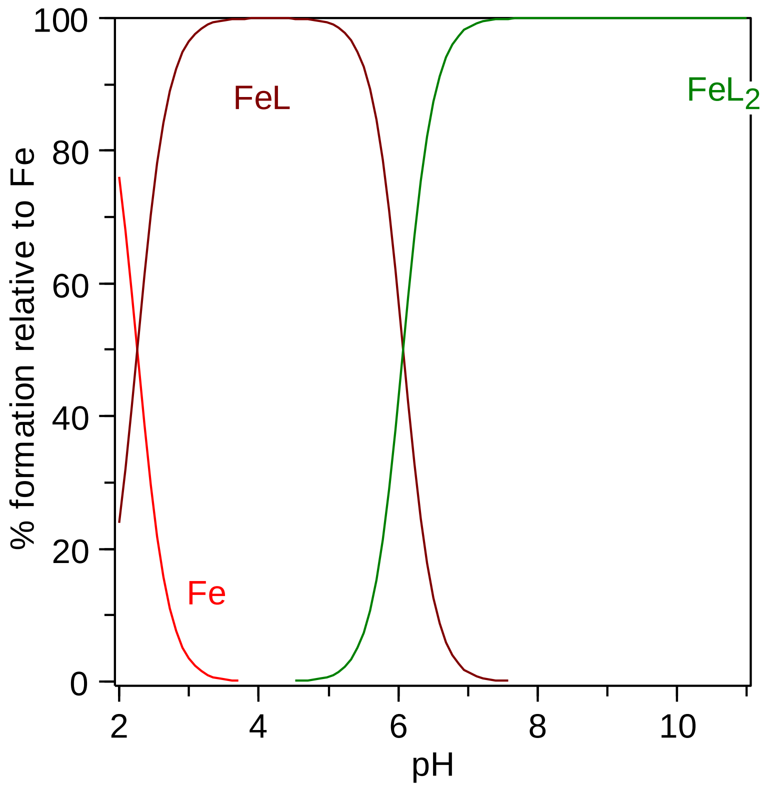

Figure S14. Distribution diagrams for the complex species of 2.

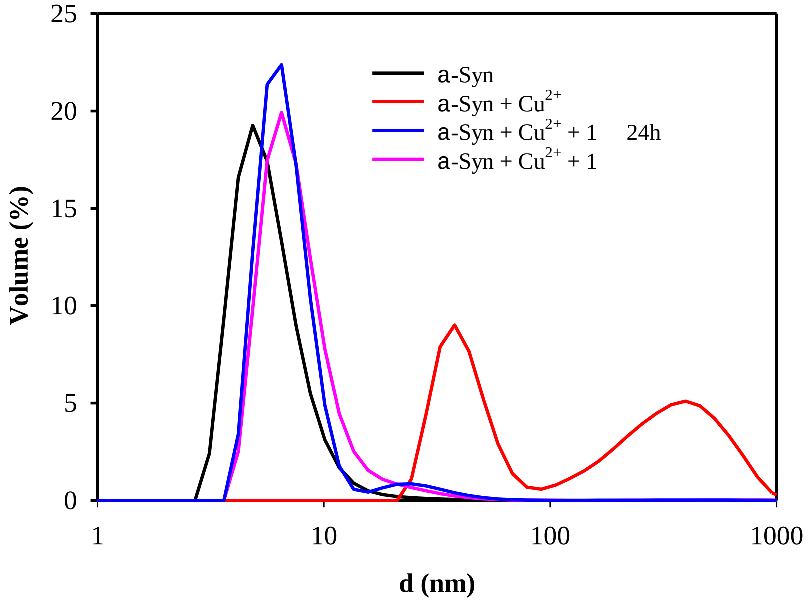

Figure S20. DLS analysis of αSyn alone and in the presence of Cu^{2+} and 1 at *t* = 0 and *t* = 24 h.

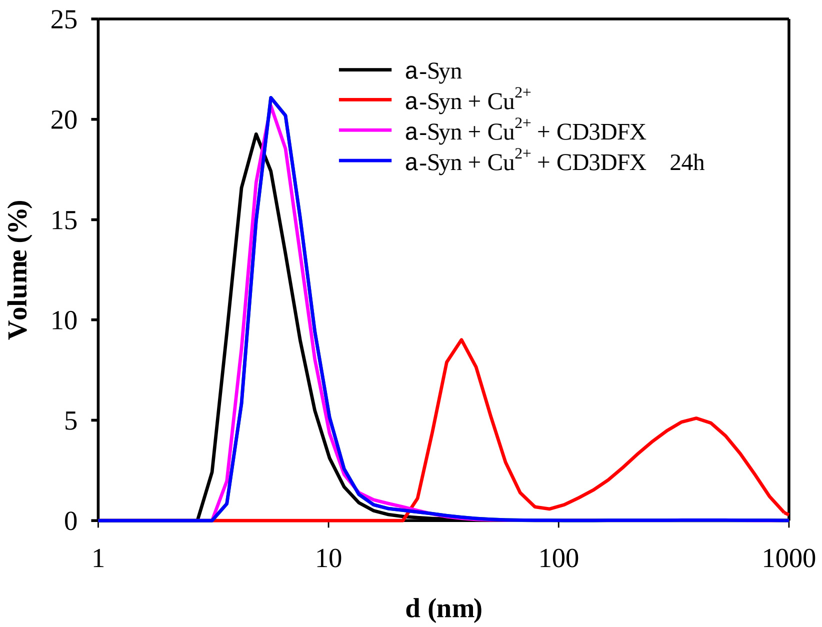

Figure S15. DLS analysis of αSyn alone and in the presence of Cu^{2+} and 2 at *t* = 0 and *t* = 24 h.

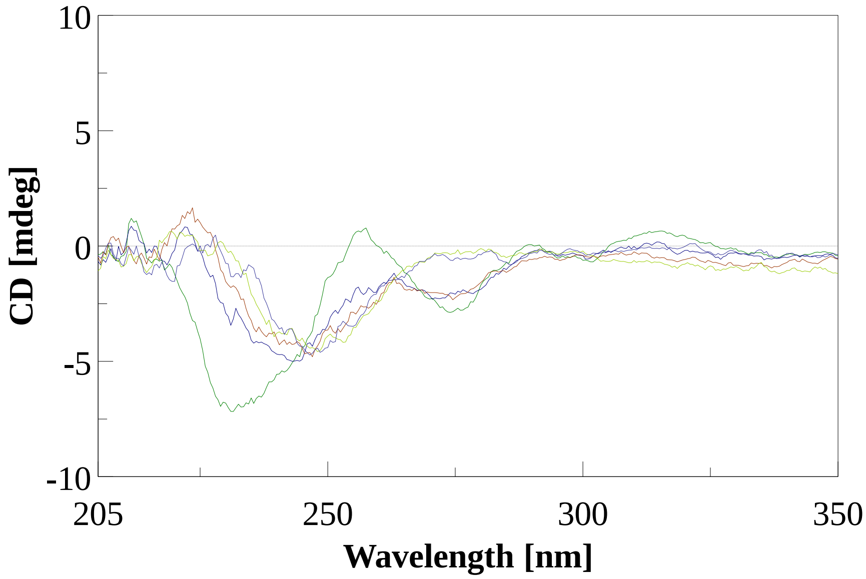

Figure S16. CD spectra of 1 (5×10^{-5} M) in the presence of increasing concentrations of Cu^{2+} (1×10^{-5} – 1×10^{-4} M).

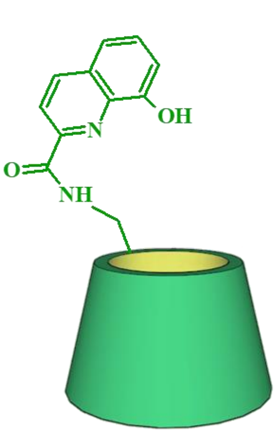

4

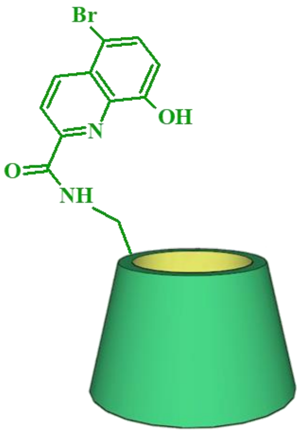

5

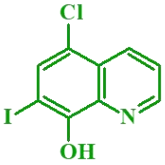

6

Figure S23. Chemical structures of the CyD-hydroxyquinoline conjugates (4 and 5) and clioquinol (6) evaluated in NPC cells for comparison.

3

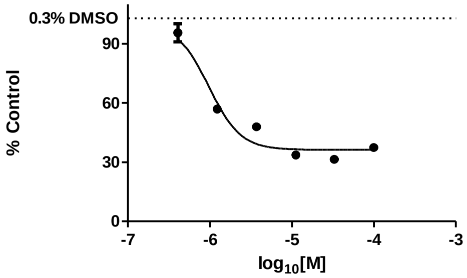

HillSlope	-2.255
IC50	9.764e-007

2

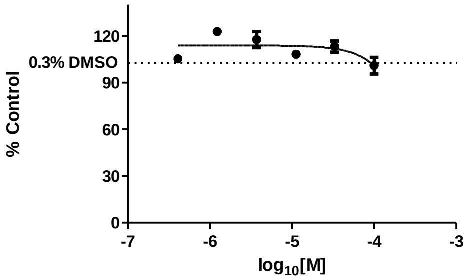

HillSlope	-1.749
IC50	0.0003250

1

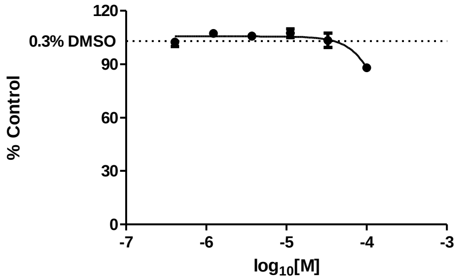

HillSlope	-2.196
IC50	0.0002076

Figure S24. HepG2 assays on 3, 2, 1. 3 duplicates, averaged.

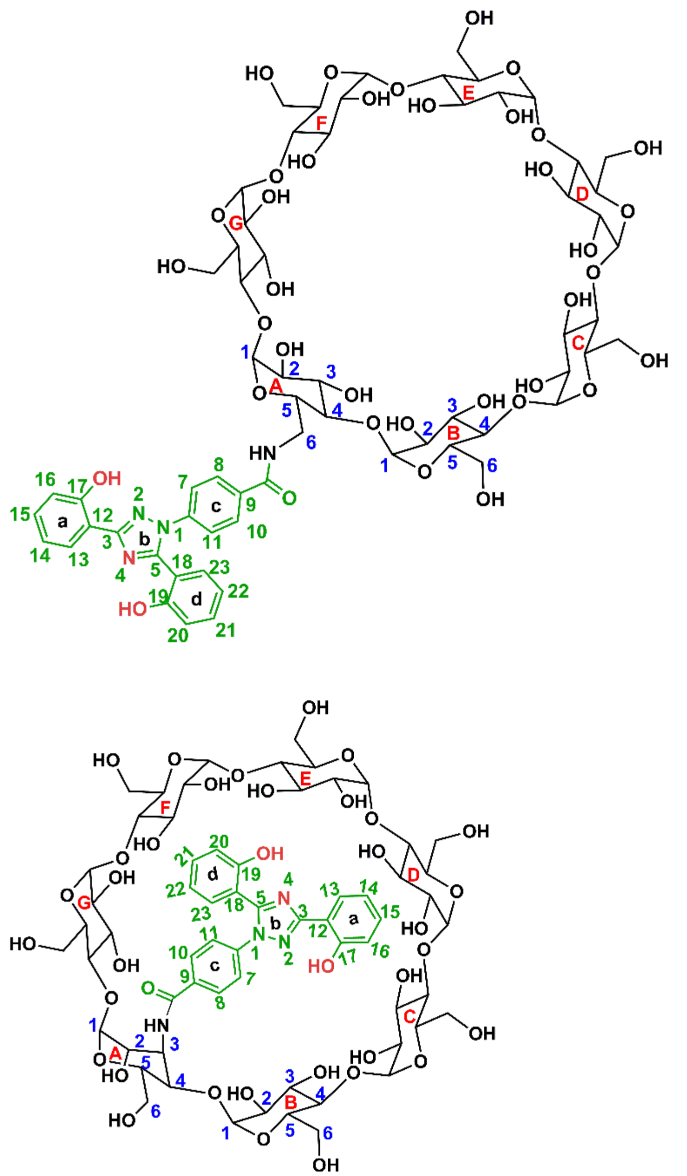

Figure S25. Nomenclature of carbon atoms of 1 and 2.

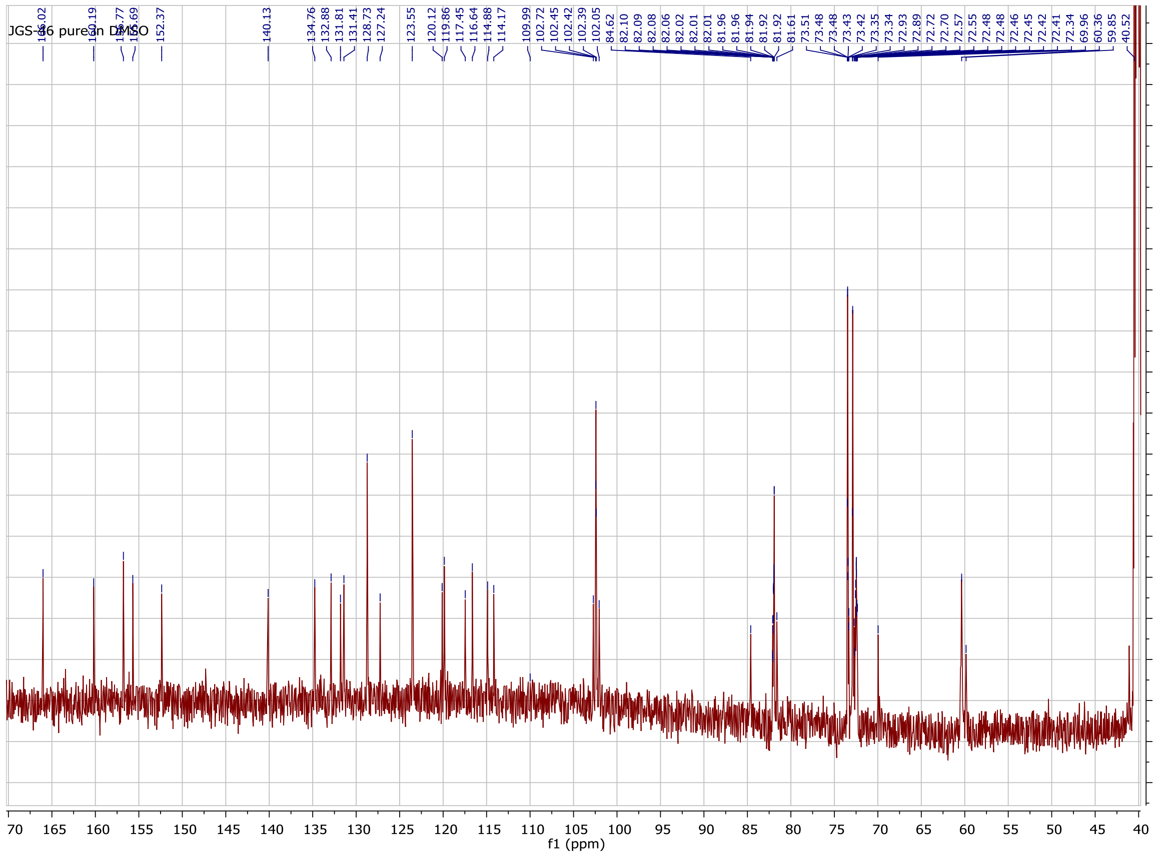
JGS-46 pure in DMSO
166.02
160.19
156.77
155.69
152.37
140.13
134.76
132.88
131.81
131.41
128.73
127.24
123.55
120.12
119.86
117.45
116.64
114.88
114.17
109.99
102.72
102.45
102.42
102.39
102.05
84.62
82.10
82.09
82.08
82.06
82.02
82.01
81.96
81.96
81.94
81.92
81.92
81.61
73.51
73.48
73.43
73.42
73.35
73.34
72.93
72.89
72.72
72.70
72.57
72.55
72.48
72.48
72.46
72.45
72.42
72.41
72.34
69.96
60.36
59.85
40.52
f1 (ppm)

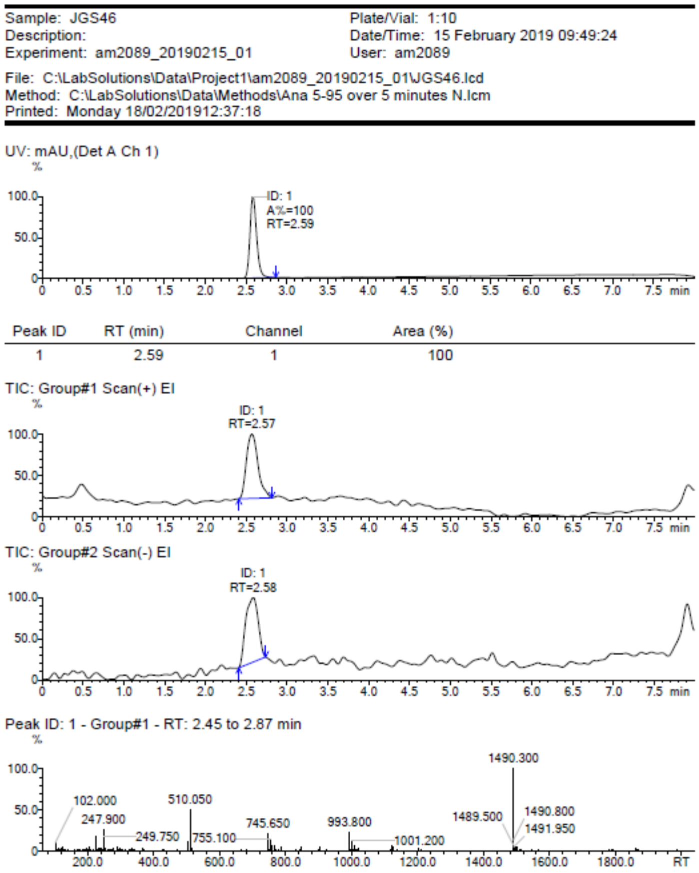

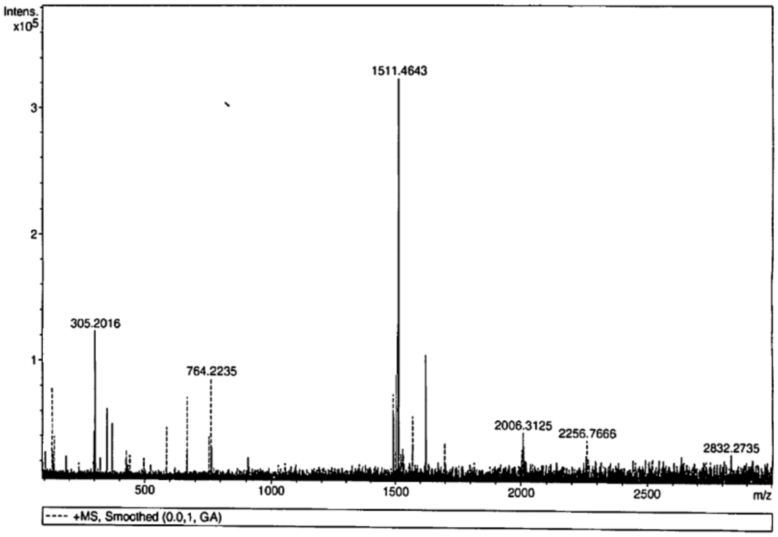

Figure S26. NMR/MS/purity of 1.

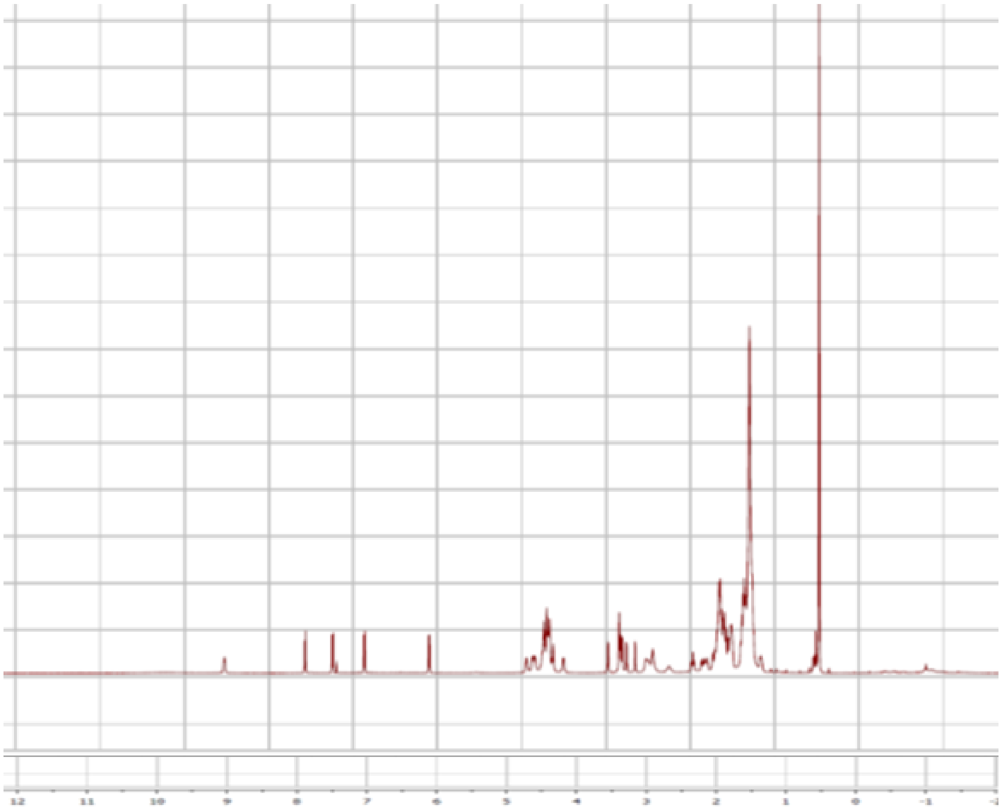
12
11
10
9
8
7
6
5
4
3
2
1
0
-1

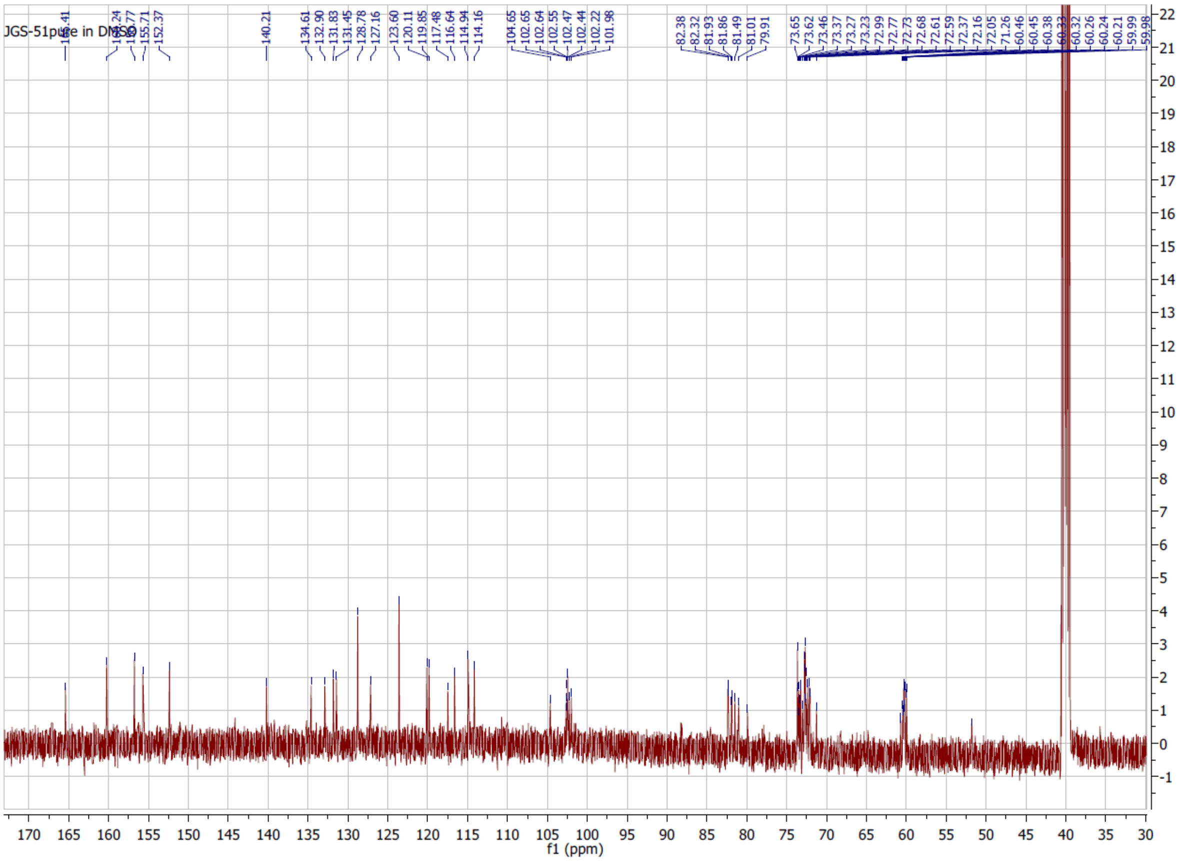
JGS-51pure in DMSO
f1 (ppm)

Sample: JGS51
Description:
Experiment: am2089_20190215_01

Plate/Vial: 1:11
Date/Time: 15 February 2019 09:59:46
User: am2089

File: C:\LabSolutions\Data\Project1\am2089_20190215_01\JGS51.lcd
Method: C:\LabSolutions\Data\Methods\Ana 5-95 over 5 minutes N.lcm
Printed: Monday 18/02/201912:38:19

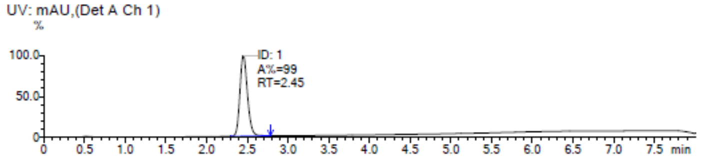

Peak ID	RT (min)	Channel	Area (%)
1	2.45	1	99.0

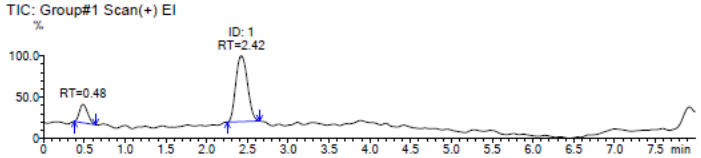

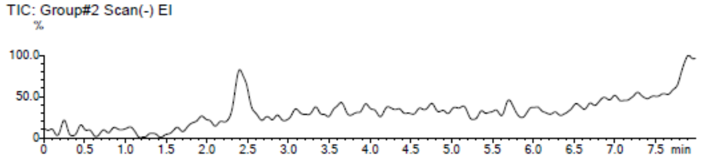

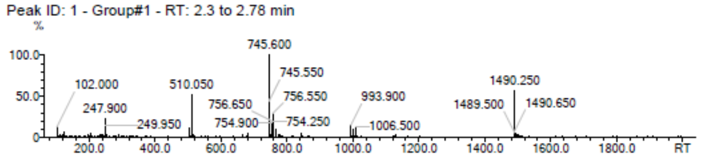

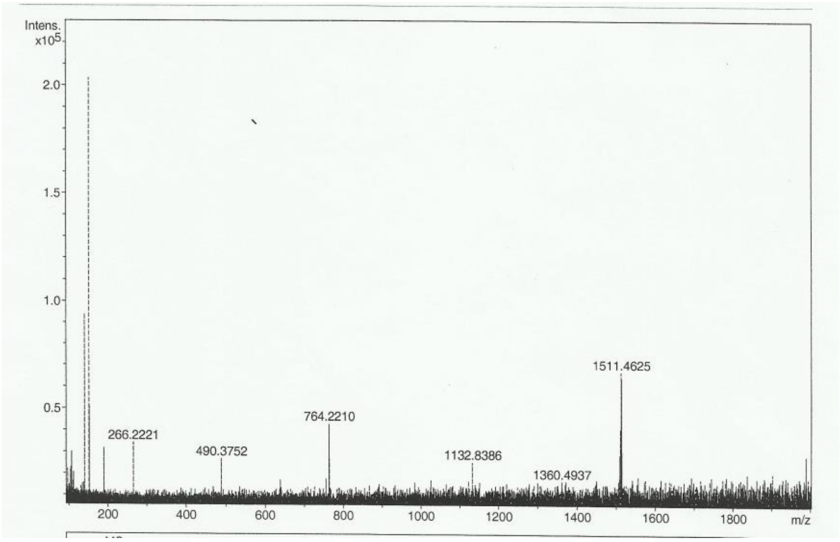

Figure S27. NMR/MS/purity of 2.

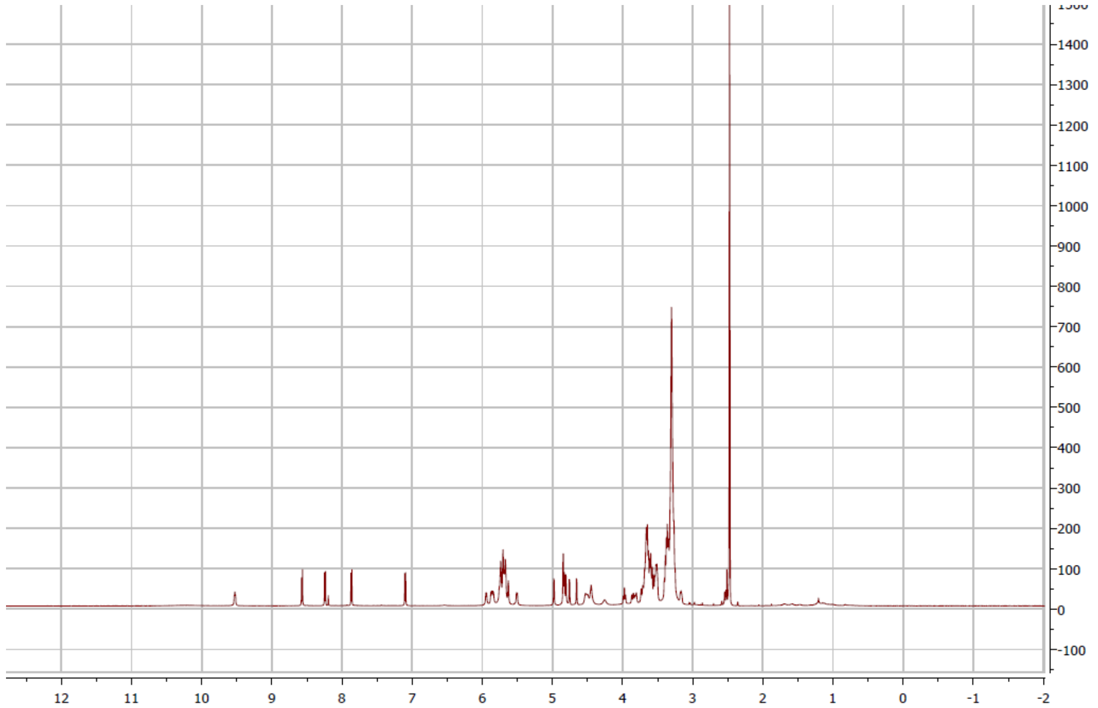

12 11 10 9 8 7 6 5 4 3 2 1 0 -1 -2
1400 1300 1200 1100 1000 900 800 700 600 500 400 300 200 100 0 -100

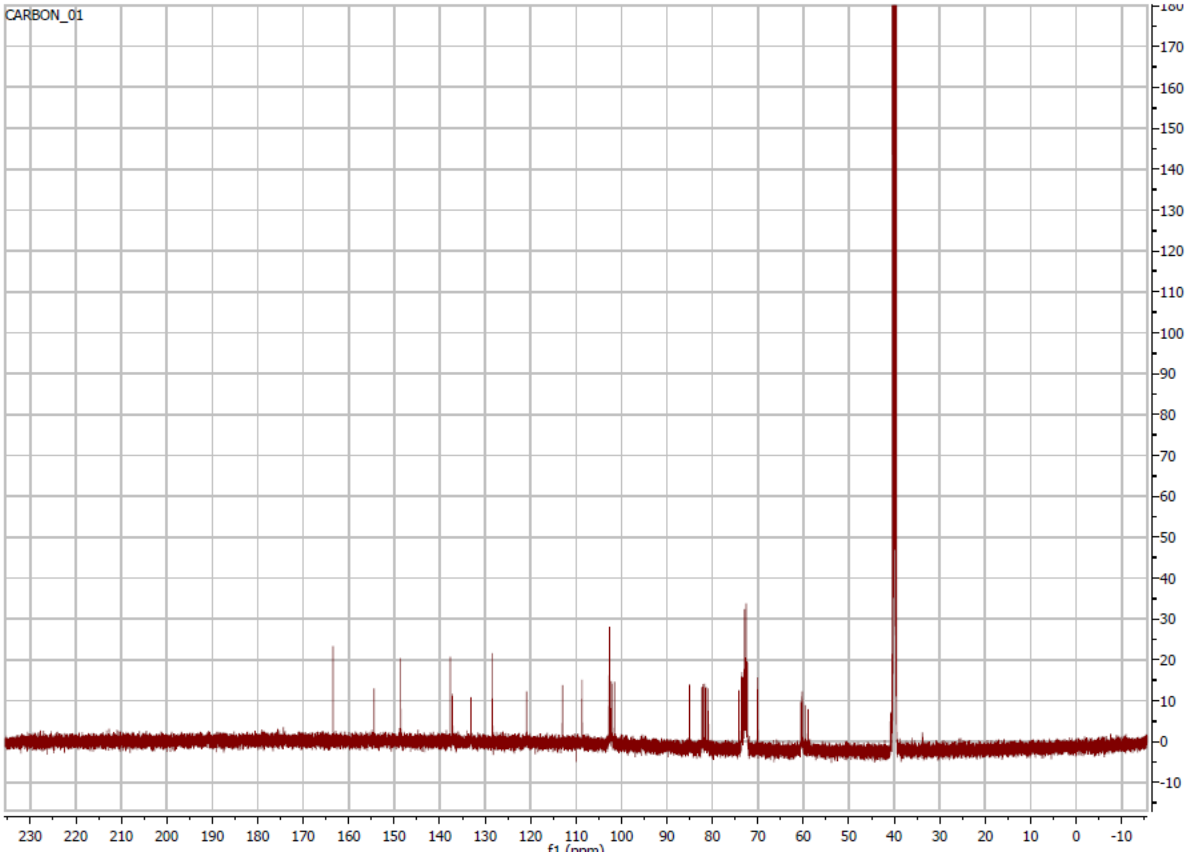

Sample: JGS139
Description:
Experiment: am2089_20190123_01
Plate/Vial: 1:25
Date/Time: 23 January 2019 12:12:52
User: am2089

File: C:\LabSolutions\Data\Project1\am2089_20190123_01\JGS139.lcd
Method: C:\LabSolutions\Data\Methods\Ana 30-95 over 8 minutes N.lcm
Printed: Thursday 24/01/201909:40:30

UV: mAU,(Det A Ch 1)

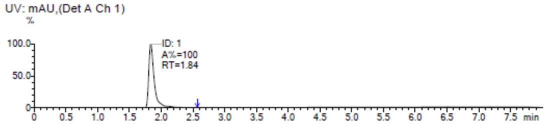

Peak ID	RT (min)	Channel	Area (%)
1	1.84	1	99.6

TIC: Group#1 Scan(+) EI

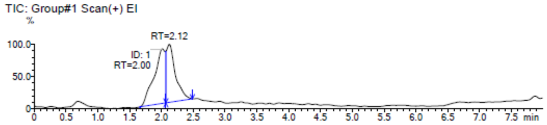

TIC: Group#2 Scan(-) EI

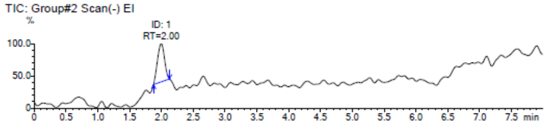

Peak ID: 1 - Group#1 - RT: 1.65 to 2.57 min

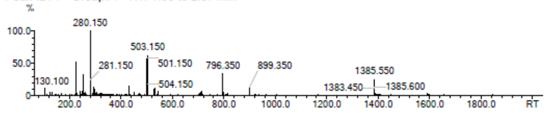

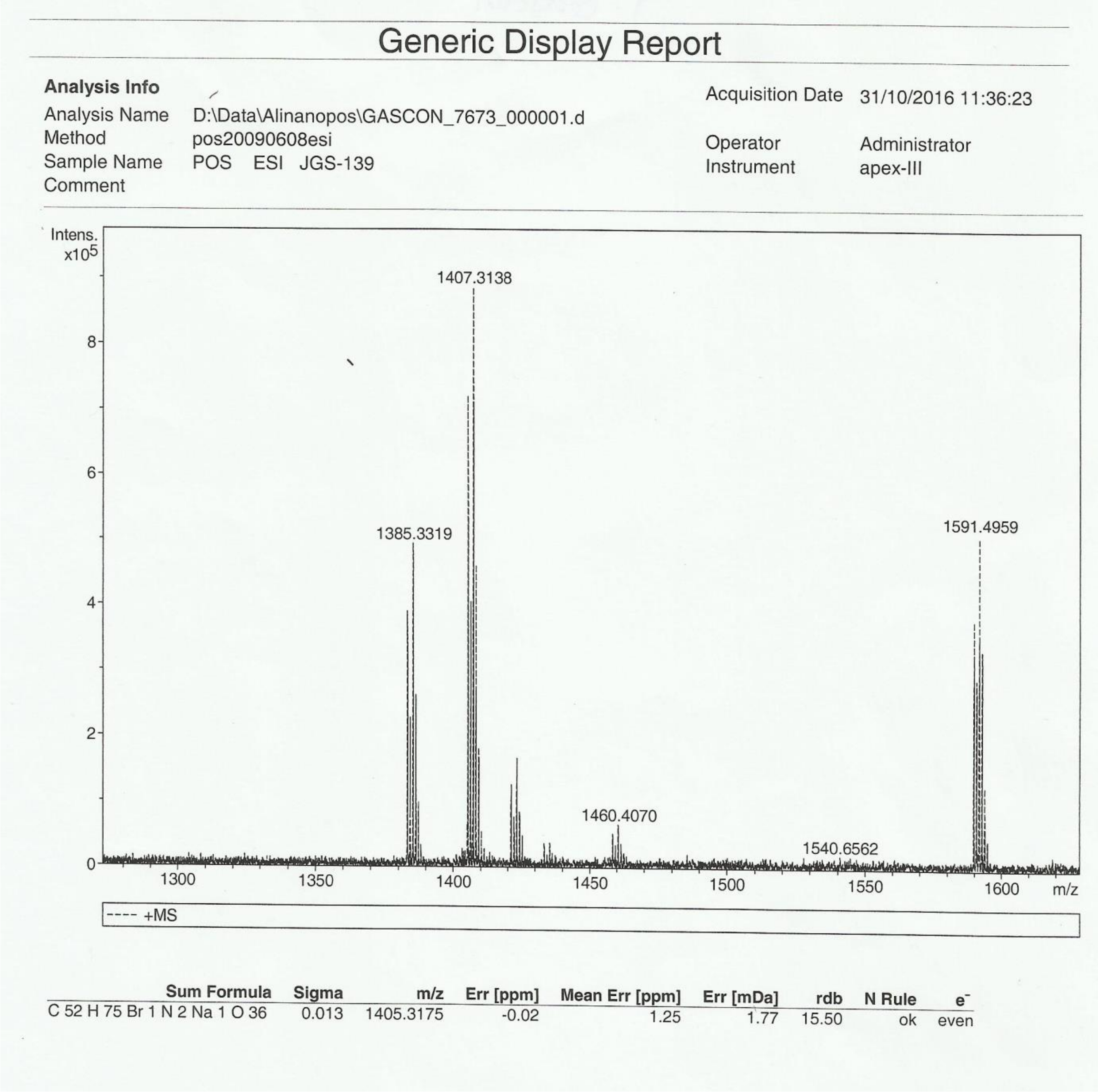

Figure S28. NMR/MS/purity of 5.

5-Bromo-8-hydroxyquinoline-2-carboxylic acid

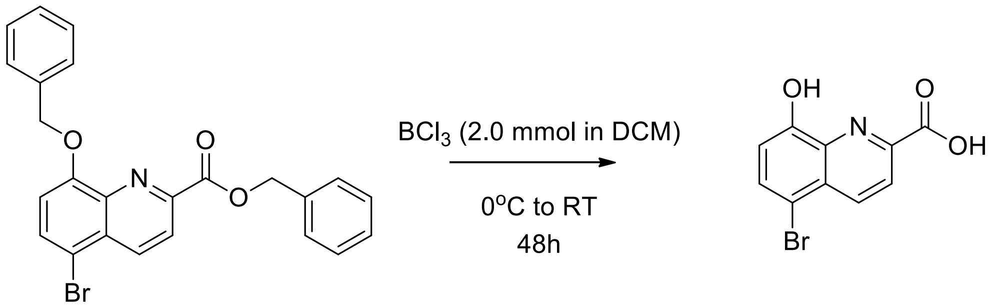

	Mw	eq.	mmol	mg	ρ	μL
benzyl 8-(benzyloxy)-5-bromoquinoline-2-carboxylate	448.31	1.0	0.21	95	-	-
BCl_3 (1.0M in DCM)	117.17	2.4	0.50			500

Benzyl 8-(benzyloxy)-5-bromoquinoline-2-carboxylate (95 mg, 1.0 eq) was suspended in DCM (10 ml) under an inert atmosphere and cooled to 0°C. To this a solution of BCl_3 (1.0 M in DCM, 0.5 ml) in DCM (1.0 ml) was added dropwise over 2 minutes.

The solution was stirred at 0°C for 5 minutes before being allowed to warm to room temperature and stirred for a further 48 hours.

After 48 hours the reaction mixture was cooled again to 0°C and additional BCl_3 (1.0 M in DCM, 2.0 ml) was added leading to a rapid colour change from colourless to a deep orange. This was stirred at 0°C for 20 minutes before being warmed to room temperature and stirred for 12 hours.

Upon completion the resulting solution was concentrated and the residual solid was triturated with hexanes (6 x 5 ml) and the mother liquid was decanted. The insoluble material was suspended in water (20 ml), collected by filtration and washed with ice cold water (2 x 10 ml) before being dried and collected as a yellow solid of 5-bromo-8-hydroxyquinoline-2-carboxylic acid (49 mg, Yield = 87 %) and ^{1}H NMR spectrum was in agreement with that in the literature.[1] Used as such.

Scheme S1. Synthesis of precursor to conjugate 5.

Table S1. Solubility data of the conjugates and controls.

Compound	solubility cut-off (μM)
Pyrene (positive control)	10<x<25
Aspirin (negative control)	>100
**1**	>100
**2**	>100
**3**	>100

References

1. **US Patent 2006/0183909 A1 (Nixon & Vanderhye, VA 22203, US). Schmitt, M. et al. Compositions Derived from Quinoline and Quinaxoline, Preparation and Use Thereof.**
2. Hider, R. *Thalassemia Reports* 2014; 4, 2261, 19-27; doi:10.4081/thal.2014.2261.
